# Surgery versus IVF for the treatment of infertility associated to ovarian and deep endometriosis (SVIDOE: Surgery Versus IVF for Deep and Ovarian Endometriosis). Clinical protocol for a multicenter randomized controlled trial

**DOI:** 10.1371/journal.pone.0271173

**Published:** 2022-08-03

**Authors:** Jessica Ottolina, Michele Vignali, Enrico Papaleo, Paola Viganò, Edgardo Somigliana, Stefano Ferrari, Valeria Liprandi, Gaia Belloni, Marco Reschini, Massimo Candiani, Paolo Vercellini, Laura Benaglia

**Affiliations:** 1 Department of Gynecology and Obstetrics, San Raffaele Scientific Institute, Milan, Italy; 2 Department of Biomedical Sciences for Health, Macedonio Melloni Hospital, University of Milan, Milan, Italy; 3 Department of Gynecology and Obstetrics, Fondazione IRCCS Ca’ Granda, Ospedale Maggiore Policlinico, Milan, Italy; 4 Department of Clinical Sciences and Community Health, Università degli Studi di Milano, Milan, Italy; PLOS: Public Library of Science, UNITED KINGDOM

## Abstract

The management of endometriosis-related infertility is still a challenging issue. Women can be managed with either surgery or in vitro fertilization (IVF). The decision is tailored to the patients considering pros and cons of both approaches. Surgery might increase the chances of natural conception and relieve symptoms. IVF may be more effective, but costs are higher and unoperated women face some peculiar additional risks during the procedure and pregnancy. The unavailability of randomized trials comparing the two strategies hampers the possibility to provide precise estimates. This Randomized Controlled Trial (RCT) aims at filling this gap. This is a multicenter, non-blinded, randomized controlled trial with parallel groups and allocation 1:1. Three Italian Academic Infertility Units will be involved. Main inclusion criteria are infertility for more than one year, age less than 40 years and a sonographic diagnosis of endometriosis (ovarian endometriomas or deep peritoneal lesions). Previous IVF and previous surgery for endometriosis are exclusion criteria. Women will be randomized to either surgery and then natural pregnancy seeking or a standard program of three IVF cycles. The primary aim is the comparison of live birth rate between the two groups (IVF versus surgery) within one year of randomization. The secondary aim is the evaluation of cost-effective profile of the two interventions. The present study can influence the clinical practice of infertility treatment in women with endometriosis. From a public health perspective, information on the more cost-effective clinical management strategy would consent a wiser allocation of resources.

**Trial registration:** NCT04743167, registered on 8 February 2021.

## Introduction

The management of endometriosis-related infertility is still a challenging issue [1–3]. Robust evidence has emerged only for the surgical treatment of superficial peritoneal endometriosis, a condition that cannot be identified without surgery [4]. However, laparoscopy to identify and treat early endometriosis is not recommended in women with unexplained infertility. According to a recent Cochrane meta-analysis, the beneficial effects of surgery are too modest to justify the procedure, at least in women without pain symptoms [4, 5].

Conversely, evidence from Randomized Controlled Trials (RCTs) is lacking for more advanced forms of endometriosis [4, 5]. These women can be managed with either surgery or *in vitro* fertilization (IVF). At present, the decision between surgery and IVF is shared and tailored to the patients taking into consideration pros and cons of both approaches and including in the discussion also the history of previous surgery, the presence of pain symptoms, women age, results of ovarian reserve testing and semen analysis. However, the unavailability of RCTs or at least robust prospective studies comparing the two strategies hampers the possibility to provide precise estimates of benefits and risks.

Surgery aims at increasing the chances of natural conception and has the beneficial effect of relieving symptoms [6, 7]. Among uncontrolled studies, the overall mean pregnancy rate is nearly 50% [8]. However, the real incremental benefit of surgery cannot be disentangled from this type of evidence, but it is presumably halved than reported in these studies [8]. Moreover, women are exposed to the risks of the intervention, including the possibility of surgery-related damage to the ovarian reserve, an effect that can hamper IVF success if natural conception does not occur after surgery [9]. To note, recent evidence demonstrated that ovarian reserve is unremarkable to natural conception provided that the remnant follicular pool is sufficient to ensure regular ovulation, i.e., up to very low level of AMH [10, 11].

IVF may be more effective, but costs are higher and unoperated women face some peculiar additional risks during the procedure and during the subsequent pregnancy. Albeit extremely rare, severe pelvic infections can occur after oocytes retrieval in women with endometriomas and pregnant women with deep peritoneal lesions may face sudden and unpredictable severe spontaneous hemoperitoneum [9].

Overall, for infertile women with endometriosis detected at ultrasound, there is a clinical equipoise that pressingly needs investigation. To provide robust evidence on this common condition, we developed a protocol for a multicentric pragmatic RCT with the primary aim of evaluating the chance of a live birth between women allocated to surgery and those allocated to IVF. In addition, as a secondary aim, the study will compare the cost-effectiveness of the two approaches. The study will include also an experimental part aimed at assessing whether the systemic inflammatory milieu of endometriosis may have a detrimental impact on the quality of folliculogenesis and embryological development. However, since this part is beyond the scope of the clinical protocol, it will be not herein described.

## Materials and methods

The hypothesis is that IVF could be more effective than surgery for the treatment of endometriosis-associated infertility in women with a sonographic diagnosis of the disease.

The study is aimed at providing robust data to be used to take clinical decision as well as drawing national and international recommendations. In particular, the aims are:

Aim 1 (primary aim): To evaluate the chance of a live birth within one year since the time of randomization between women allocated to surgery and those allocated to IVF.

Aim 2: To compare the cost-effectiveness of the two approaches using the health care system perspective.

### Setting

This study is a multicenter, non-blinded, randomized controlled trial with parallel groups and allocation 1:1. Three Italian academic infertility units will be involved:

1. Fondazione IRCCS Ca’ Granda, Ospedale Maggiore Policlinico, Milan, Italy.
2. IRCCS Ospedale San Raffaele, Milan, Italy.
3. ASST-FBF-Sacco, Presidio Ospedaliero Macedonio Melloni, Milan, Italy

### Participants

Inclusion criteria are as follows:

- Age < 40 years
- Pregnancy seeking for more than 12 months
- Regular menstrual cycle, i.e., mean cycle interval between 21 and 35 days
- Ultrasonographic diagnosis of ovarian endometriomas or deep peritoneal endometriosis.
- Normal seminal analysis based on WHO criteria [12]
- Absence of ureteral stenosis or intestinal subocclusive symptoms.

Exclusion criteria are as follows:

- Previous surgery for endometriosis
- Previous IVF cycles
- Contraindication to pregnancy
- Hydrosalpinx
- Endometriomas with a mean diameter > 4 cm
- Submucosal fibroids or large intramural or subserosal fibroids (> 4 cm).
- Doubtful sonographic findings that do not allow to reliably rule out malignancy.
- Obstacles to regular sexual intercourses (sexual disturbances or logistic problems)

The ultrasound diagnosis of endometriosis will be performed according to international standards [13]. The diagnosis of endometrioma will be done according to these established imaging criteria [13] and, in addition, it will have to be documented on at least two occasions and at least two menstrual cycles apart. Deep nodules visualized at ultrasound in proximity with the uterine cervix, or behind the cervix (posterior compartment) or within the bladder wall (anterior compartment) will be also recorded [13]. Broad ligaments with particular attention to distal ureters will also be routinely investigated to rule out stenosis. The dimensions of the nodules and cysts will be measured in three orthogonal planes [13]. Finally, the presence of adenomyosis will also be recorded and described [14]. Affected cases will not be operated to remove the lesions or treated differently. The presence of this anomaly will not affect allocation but will be used at the end of the study in the interpretation of the findings.

Severe pain symptoms will not be an exclusion criterion. Women with these symptoms allocated to IVF will be firstly treated with hormonal treatment (progestins, estroprogestins or GnRH analogues) to manage their pain prior to embark in IVF. These treatments will be transiently discontinued only during the IVF attempts. In case of pain-resistance to hormonal treatment, the woman will be scheduled to surgery but maintained in the IVF arm (intention to treat analysis).

### Interventions

#### Primary aim

Women accepting to enter the study will be randomized to either surgery and then natural pregnancy seeking or a program of three complete IVF cycles (i.e., three oocyte retrievals regardless of the subsequent number of embryo transfers that will be possible). Both approaches will be performed according to local standards. The initial time point will be the time of randomization. Eligible patients will undergo a first visit of screening and an accurate transvaginal ultrasound assessment according to international standards [13]. Women eligible for the study will be referred by care providers to a member of the research team who will describe the informed consent process. Details of the study will be explained to patients, including its main aims, procedures, temporal commitment, possible discomforts and risks, benefits. Recruitment will be on a voluntary basis with the right to withdraw from the study at any time; moreover, women will be informed that the decision to join the study protocol will not affect their possibility to shift to the other technique (surgery or IVF). Finally, informed consent will be illustrated and provided to the patients willing to participate. Operators will give patients the opportunity for questions and enough time to consider their participation. After two weeks, patients who will agree to enter the study will refer again to return the signed consent and to be randomized. During this visit, demographic and clinical characteristics as well as ultrasound findings will be recorded.

Randomization will be organized centrally by Redcap (version for Fondazione IRCCS Ca Granda Ospedale Maggiore Policlinico, Milano: https://redcap.policlinico.mi.it/). The allocation sequence will be computer-generated. The allocation ratio will be 1:1. Randomization list will be stratified for the three participating centres. All patients, all caregivers and embryologists at the clinical departments will not be blinded to trial intervention allocation after inclusion. The progress of the study will be periodically monitored by an external monitor to verify the strictness of the data management.

Once randomized, both patients and physicians will not be blinded to the treatment arm. Women of both study groups will initiate treatment (surgery or IVF) in a shortest delay, maximum 3 months. Women undergoing surgery will be investigated for tubal patency during surgery for endometriosis throughout salpingocromoscopy. After surgery, patients allocated in this arm, will be informed about their chances of natural pregnancy using the Endometriosis Fertility Index (EFI) questionnaire [15]. They will be monitored with ultrasound scan every three months and suggested to timing sexual intercourses using LH urinary tests.

**Fig 1:**
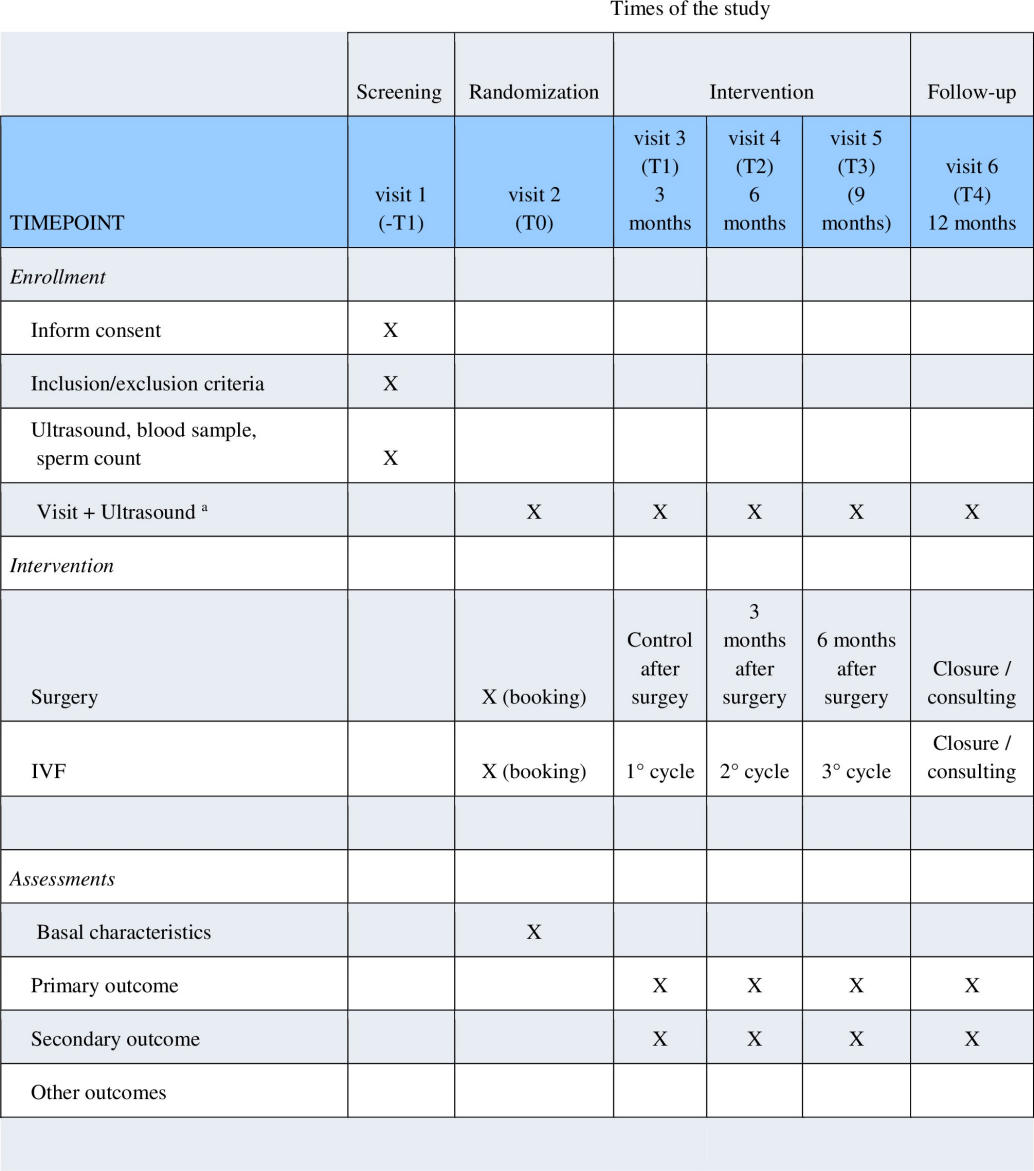
A schedule of enrolment, interventions and assessments of the study protocol.

Patients allocated in IVF arm will undergo three complete IVF cycles (i.e. three oocyte retrievals regardless of the number of embryo transfers performed). Women will be managed according to a routine clinical protocol as reported elsewhere [16–18]. Pre-procedure aspiration of the endometrioma is not part of the local policies of the participating centers and will not be performed. Surgery will be achieved according to local standards [5, 6, 19]. No efforts were done to standardize the surgical modalities. Given the pragmatic design, we favored the expertise to the standardization. The random allocation stratified per center is expected to overcome the possible impact of technical differences. Visits in both arms will be done every 3 months. Patients will be controlled about symptoms or new endometriotic lesions throughout ultrasound scans. Final visit will take place 12 months from randomization. If pregnant, women will be contacted even later to assess the evolution of pregnancy. A schedule of enrolment, interventions and assessments of the study protocol is reported in Fig 1. The full protocol is included in S1 Protocol.

Even if patients will be informed and made aware about the importance of persisting in the allocated arm because inefficacy of treatment cannot be drawn prior to complete the whole course (surgery + 9–12 months of natural pregnancy seeking versus three completed IVF cycles), they will be allowed to switch to the alternative treatment at any time (pragmatic trial). Drop-out from the IVF program without undergoing surgery is another possibility that will not be a reason for exclusion. However, for the analyses, all these cases will remain in the originally allocated arm (intention to treat). The ultimate aim is comparing the success at one year between the decision to initiate with surgery or to initiate with IVF, regardless of the subsequent trajectories of the included subjects. A per protocol analysis will also be done, but this is not the primary intent. Reasons surrounding the decision to switch will be recorded. At the end of the study period (12 months), women failing to conceive will be counselled about the option to persist in the allocated arm versus a different treatment: i.e., women who will be allocated to surgery will be offered IVF and the other way around for those allocated to IVF.

Given the uncertainty regarding the willingness to participate to a RCT comparing two radically different approaches, we will propose to women refusing randomization to participate to a parallel study that will assess the same outcomes as those who will be randomized, even if the decision is taken by patients rather than being randomly sorted (*patient preference trial*).

Any adverse event will be promptly communicated to local ethics committees. The adverse events that may occur are those related to surgery or to the IVF cycle and will not differ from what is expected in normal clinical practice.

Specific training for participant clinicians is not required because the studied interventions are part of normal clinical practice in the participating centers. All three groups are academic and offer the whole spectrum of required expertise, from sonographic diagnosis to surgical or IVF treatments. To note, members of all involved units are also part of the Endometriosis Treatment Italian Group (ETIC), an independent Italian group of free thinkers that regularly meet to discuss aspects of endometriosis management [5]. However, two preliminary meetings for all the personnel of the participating units prior to initiate were done to discuss in depth the protocols.

Sample size calculation was done by Fleiss method with continuity correction and was based on the following assumptions: 1) expected success rate in the surgical group: 30%, 2) type I and II errors of 0.05 and 0.20, 3) difference in favour of IVF justifying the additional costs of the procedure of 20% (absolute rate of success of 50%). On these bases, the number of women to be randomized is 206 (103 per arm). Considering an expected rate of eligible women declining treatment of about 30%, the total number of women to be initially selected would be 300.

### Secondary aim

The economic analyses will be performed using the public health system perspective by applying the reimbursements used in Lombardy Region, Northern Italy. Costs of hospital care will be obtained using local tariffs of diagnosis related groups (DRG). Costs of drugs will exclusively include those that are reimbursed by the public health system (mainly gonadotropins). Costs of infertility assessments will not be included since considered similar in the two arms of the study. In contrast, we will include additional costs needed to handle complications of surgery or IVF in the two arms as well as those required for obstetrics management of complications (including neonatal assistance). Specifically, we will check the patients at T1, T2, T3 and T4 to register costs of hospital care, costs of drugs and additional costs needed to handle complications of surgery or IVF in the two arms. Finally, we will contact any pregnant woman to include costs for obstetrics management of complications (including neonatal assistance). Indeed, we cannot exclude that some differences in pregnancy course between the two arms of the study could emerge. The main outcome will be the costs per live birth.

### Data collection and analysis

Data will be analysed using the Statistical Package for Social Sciences (SPSS 26.0, IL, USA). The analyses will be performed by intention to treat. An interim analysis is not planned. However, rate of recruitment will be strictly monitored to identify early possible obstacles and, if possible, to overcome them. Recruitment phase is planned to be 18 months (but may last 12 additional months if needed). Regarding the primary outcome, the live birth rate, no adjustment are planned and the result will be presented as crude Relative Risk (RR) and 95% Confidence Interval (95%CI). Data will be reported as mean ± standard deviation (SD), median [interquartile range-IQR] and number (%) and will be compared using Student *t* test, nonparametric Mann-Whitney test, Chi squared test or Fisher Exact test, as appropriate. Shapiro-Wilk test will be preliminary performed to assess the consistency of the data with normal distribution. P values below 0.05 will be considered statistically significant.

The primary outcome (reported as crude Relative Risk (RR) and 95% Confidence Interval (95%CI) will be calculated considering also the following pre-specified subgroups: study center, ovarian reserve status (two groups based on median levels of AMH), age (< and > 35 years), semen analysis (two groups based on median levels of the total number of motile spermatozoa), type of lesions (endometriomas, deep lesions or both) and pain symptoms without medical therapy (at least one symptom with numeric rating scale > 5 versus none). If a high number of patients will not accept randomization, an analysis of the outcome will be performed considering separately these women (*patient preference trial*). We will perform the same analyses scheduled for the RCT. However, the chances of live birth will be adjusted for age and baseline variables found to differ between the study groups (with a p value below 0.10).

Recruited patients will be assigned anonymous codes and data analysis will be performed on anonymized datasets. Patients’ data collected during the study protocol will be treated in accordance with the Italian 196/2003 Data Protection Act. Electronic datasets will be accessed through a personal password; paper documentation will be accessible only to study investigators. Study data will be kept for at least 10 years after publication of study results.

An active follow-up after the end of treatment will be performed with own local resources. Indeed, once terminated women who will not achieve pregnancy will be offered surgery for those allocated to IVF and IVF for those scheduled to surgery. Two additional years will be needed to obtain these results, but we deem important presenting them to the scientific community. They may provide a more complete overview of the problem since, in clinical practice, this type of approach (shifting from one option to the other in case of failure) is common.

### Status and timeline of the study

The study protocol was approved by Ethical Committee Area 2 Milan (approval number 0001332) on 12th January 2021, by Ethical Committee IRCCS San Raffaele Hospital (approval number 55/2021) on 10th March 2021 and by Ethical Committee Area 1 Milan (approval number 0024516) on 12th May 2021. All participating women will sign a written informed consent, as approved by the Ethical Committees. In the event of any important modification to the study protocol, the principal investigator (L.B.) will inform study team members and trial participants via email/phone or in person.

The enrollment of patients is ongoing. Timeline of the enrollment of patients will be March 2022. The end of the study will be in December 2024.

## Discussion

The management of endometriosis in women with infertility remains controversial [1, 2]. Robust evidence for severe endometriosis is lacking. To date, there are neither RCTs, nor prospective comparative studies aiming at clarifying the potential benefits of surgery versus IVF in women with more advanced endometriosis, specifically, those carrying ovarian endometriomas or deep peritoneal lesions identifiable at ultrasound. The present study can potentially influence the clinical practice of infertility treatment in women with these forms of endometriosis.

This RCT requires a significant and coordinated effort but the results will be outstanding for the scientific and clinical national and international community, regardless of which treatment will prove to be more effective or more cost-effective. For the first time, physicians and patients will have a clear overview of the effectiveness of the two approaches and could take more informed decisions. In addition, public health stakeholders will have precious information useful to base their recommendation or decide refundability. Endometriosis is not rare among infertile women (15–30%) [1]. From a public health perspective, information on the more cost-effective clinical management strategy would allow a wiser allocation of resources and a reduction of wastages.

Reasons to explain the lack of robust evidence on this topic in the literature are complex and an in-depth discussion of this point is beyond the scope of this manuscript. Nonetheless, we hypothesize that a crucial role was played by the long-lasting and still not fully overcome belief that histological confirmation (and therefore surgery) is essential for a definitive diagnosis of endometriosis. This vision is now out-dated [5]. Gynaecological ultrasonography underwent impressive progresses over the last two decades and more and more endometriosis centres now offer the opportunity to perform advanced sonography. According to a recent Cochrane review, transvaginal ultrasound for endometriomas had a sensitivity of 0.93 (95%CI: 0.87–0.99) and a specificity of 0.96 (95%CI: 0.92–0.99). For deep peritoneal lesions, sensitivity and specificity are 0.79 (95%CI: 0.69–0.89) and 0.94 (95%CI: 0.88–1.00), respectively [20]. To note, in the context of the present study, specificity is more important than sensitivity and, for both type of lesions, this parameter is actually optimal.

The study presents some limitations and challenges that deserve to be commented. The most important are the following.

Firstly, recruitment and adherence may be a concern. The two strategies radically differ and, even if there is a scientific equipoise, patients may be reluctant to accept random allocation. A preliminary study of our group already highlighted this possible difficulty [21]. For this reason, women declining randomization but agreeing to participate will be maintained in the study and monitored (*patient preference trial*). To note, prospective comparative studies on the studied issue are absent in the literature and we estimate that we could provide valuable information also in case of failed recruitment for the RCT. In addition, we *a priori* postulated a high rate of declines (about one out of three) and we aim to identify 300 eligible women. Sensitivity analyses on sample size justification showed that reducing the total sample size up to 130 women (65 per arm) could also provide valuable information for a difference of success between the two arms of at least 25% (with the same study power). In other words, the study could be interesting even if the recruitment will be reduced by 37% (from 206 to 130).

A second possible concern is the non-adherence to the scheduled treatment for the whole study period. For this reason, we decided to analyse data by intention to treat. As such, deviations (leaving natural pregnancy seeking after surgery earlier or deciding for surgery prior to complete the three IVF attempts or drop-out from IVF program regardless of the decision to perform surgery) will not justify exclusion. In fact, after surgery, women will be also counselled using the EFI index and, on this basis, they may also decide to shift to IVF earlier. Somehow, even if less pure, evidence obtained with our study will better stick to everyday clinical practice.

Thirdly, involved clinicians may be reluctant to let their patients participate to the RCT. A main possible cause of reluctance to participate or deviation could be age and ovarian reserve testing. Indeed, there is an unproven tendency to over-estimate the detrimental effects of timing on the chances of IVF. This caused a general trend to prematurely rush to IVF. Age and ovarian reserve are crucial for IVF success but the impact of a one-year delay is modest. For instance, in a recent theoretical model, we highlighted that even in women older than 35 years, a 6-months delay in the access to IVF is unremarkable [22]. The rate of success of IVF linearly declines after age 35, with an absolute loss of 2–4% per year [22]. This loss seems unremarkable if balanced with the expected benefit of surgery of 30%. In addition, recent evidence clearly showed that a low AMH cannot be used to infer a more rapid decline in ovarian reserve [23], thus questioning the common belief that low AMH is an indication to anticipate IVF. Noteworthy, one may also speculate that surgery could be even wiser in women with reduced ovarian reserve. The amount of residual ovarian reserve does not affect the chances of natural pregnancy [24] and a low ovarian reserve testing was shown to be poorly predictive of premature menopause [23]. In other words, a clinical equipoise persisted also in women with low ovarian reserve, and, for this reason, these subjects were not excluded. Even if women with this condition are commonly scheduled to IVF because of the fear that surgery could further damage ovarian reserve, the incremental benefit of IVF over surgery may conversely be narrower. A specific seminar dedicated to the complex relation among age, ovarian reserve, natural pregnancy, and IVF success was organized prior to initiate recruitment for all physicians who are engaged in the study to prevent undue beliefs that could ultimately be deleterious for recruitment.

The presence of endometriosis does not necessarily mean that the disease is the origin of infertility. Other coexisting causes can explain the incapacity to conceive. This is an additional possible concern. Two points merit to be highlighted here. First, we will exclude women with irregular menstrual cycles or those whose partner has abnormal semen (see inclusion criteria). This is expected to exclude women with disovulatory dysfunctions and those with a male cause of infertility. Moreover, in the exclusion criteria, we have listed the presence of hydrosalpinx at ultrasound. This is expected to exclude women with other severe causes of tubal infertility. To note, pre-inclusion assessment of tubal patency was not deemed necessary given the invasiveness of the tests and the scant clinical relevance. Indeed, in women scheduled to IVF, tubal patency is not clinically interesting while in those scheduled to surgery this aspect will be investigated in the theater and, if needed, tubes will be concomitantly treated. Second, this is a pragmatic study. Therefore, the inclusion of some women with endometriosis whose real cause of infertility is not the disease itself is possible but does not affect the clinical relevance of the trial. In clinical practice, endometriosis is deemed causative (and treated) when the disease is documented at ultrasound in couples whose infertility work-up is otherwise unremarkable.

Finally, one may disagree on the exclusion of women carrying endometriomas larger than 4 cm. This choice is linked to the evidence that large cysts impact on the clinical equipoise on which the study is based. Indeed, the presence of endometriomas with a mean diameter above 4 cm significantly decreases the number of oocytes retrieved [25, 26]. Moreover, large cysts may be associated to severe pain and surgery is highly effective in attenuating symptoms in these cases. Overall, for endometriomas larger than 4 cm, the clinical balance tips more clearly in favor of surgery [27] and, for this reason, we will exclude these cases. In this regard, one should also underline that pre-IVF aspiration of endometriomas will not be done. This policy was decided because of the risk of infection. Our trial will not be able to draw inference on the possible beneficial effects of this intervention and further evidence will be needed to address this aspect, in particular for large endometriomas.

Overall, despite the above-mentioned limitations and challenges of our study design, we believe that this RCT could provide clinically relevant findings, regardless of which treatment will prove to be more effective or more cost-effective. In fact, considering the medical point of view, it can influence clinical practice in the management of infertile women with severe endometriosis. On the other hand, from an economic perspective, information on the more cost-effective clinical management strategy would allow a wiser allocation of resources and a reduction of wastages.

## Supporting information

S1 Checklist SPIRIT 2013 checklist: Recommended items to address in a clinical trial protocol and related documents*. (DOCX)

S1 Protocol Original full protocol. (PDF)

S2 Protocol Original full protocol. (DOCX)

## List of abbreviations

IVF: In vitro fertilization
EFI: Endometriosis Fertility Index
EV: Extracellular Vescicle
NTA: Nanoparticle tracking analysis
DRG: Diagnosis Related Group
CI: 95% confidence interval
LBR: Live birth rate
RCT: Randomized controlled trial
SVIDOE: Surgery Versus IVF for Deep and Ovarian Endometriosis
