# Supplementary material for: Surgery versus IVF for the treatment of infertility associated to ovarian and deep endometriosis (SVIDOE: Surgery Versus IVF for Deep and Ovarian Endometriosis). Clinical protocol for a multicenter randomized controlled trial
Source: PLoS One. 2022 Aug 3;17(8):e0271173. doi: 10.1371/journal.pone.0271173 (PMC9348732; doi:10.1371/journal.pone.0271173)
Supplement: S1 Protocol — (PDF) [file pone.0271173.s002.pdf]

IVF versus surgery for the treatment of infertility associated with ovarian and deep endometriosis.

SVIDOE (Surgery Versus IVF for Deep and Ovarian Endometriosis)

Protocol version 1.0 (12/11/2020)

Promoter: Fondazione IRCCS Ca' Granda Ospedale Maggiore Policlinico,
Via Sforza 28, 20122 Milan, Italy

P.I. Laura Benaglia M.D.

P.I. Satellite Centre Jessica Ottolina M.D.

1. INTRODUCTION

Background / State of Art

The management of endometriosis-associated infertility remains controversial (Vercellini *et al*.,2014; Somigliana *et al*., 2017; Hodgson *et al*., 2020). Robust evidence has emerged only forsuperficial peritoneal endometriosis, a situation that cannot be identified without surgery. According to a recent Cochrane meta-analysis, odds ratio (OR) of viable intrauterine pregnancy in women undergoing laparoscopic treatment for superficial peritoneal endometriosis compared to diagnostic laparoscopy is 1.89 (95%CI: 1.25-2.86) (Bafort *et al*., 2020). Despite this evidence, performing diagnostic laparoscopy in all women with unexplained infertility to identify and treat those with early stage endometriosis is questionable. The absolute increase in pregnancy is modest and, since endometriosis is present in only 30-50% of eligible women, at least half of the patients are uselessly exposed to the risks and costs of surgery. Overall, the value of care is low and diagnostic laparoscopy in women with unexplained infertility to identify and treat superficial peritoneal endometriosis is currently not recommended (ETIC, 2019).

Robust evidence for all the other forms of endometriosis is lacking. To date, there is no RCTs neither prospective comparative studies aimed at clarifying the potential benefits of surgery versus assisted reproductive techniques in women with more advanced endometriosis, in particular those carrying ovarian endometriomas or deep peritoneal lesions. Of interest here is that, in contrast to superficial peritoneal endometriosis, these forms of the disease can now be reliably diagnosed without the need of surgery. Transvaginal ultrasound in particular is easily available and considered highly accurate. According to a recent Cochrane meta-analysis, sensitivity and specificity of transvaginal ultrasound for the detection of ovarian endometriomas are 0.93 (95%CI: 0.87-0.99) and 0.96 (95%CI: 0.92-0.99), respectively (Nisenblat *et al*., 2016).

For deep peritoneal endometriosis, they are 0.79 (95%CI: 0.69-0.89) and 0.94 (95%CI: 0.88- 1.00), respectively (Nisenblat *et al*., 2016). In addition, the modalities for diagnosis are now standardized and shared in the scientific community (Guerriero *et al*., 2016). In fact, surgery is no more considered essential for diagnosis. This recent recognition of the validity of the non-invasive diagnosis of advanced endometriosis represents an outstanding achievement and a cornerstone for a wiser management of the disease. Infertile women with endometriosis detected at ultrasound now face two therapeutic alternatives, surgery or *in vitro* fertilization (IVF). Surgery aims at increasing the chances of natural conception but the magnitude of this benefit remains controversial and women are exposed to the risks of the intervention, including the possibility of surgery-related

damage to the ovarian reserve (Somigliana *et al.*, 2015). IVF may be more effective, but costs are higher and unoperated women face some peculiar additional risks during the procedure and during the subsequent pregnancy.

Albeit extremely rare, severe pelvic infections can occur after oocytes retrieval in women with endometriomas and pregnant women with deep peritoneal lesions may face sudden and unpredictable severe spontaneous hemoperitoneum (Somigliana *et al.*, 2015). At present, the decision between surgery and IVF is shared and tailored to the patients taking into consideration pros and cons of both approaches and including in the discussion also the history of previous surgery, the presence of pain symptoms and effectiveness of medical treatment, women age, results of ovarian reserve testing and semen analysis. However, the unavailability of RCTs or at least robust prospective studies comparing the two strategies hamper the possibility to provide precise estimates and, not surprisingly, management drastically differ among centres.

2. OBJECTIVE/HYPOTHESIS

In this study, we hypothesise that IVF could be more effective than surgery for the treatment of endometriosis-associated infertility in women with a sonographic diagnosis of the disease. The study is a non-blinded, randomized controlled trial. It is aimed at providing robust data to be used to take clinical decision as well as drawing national and international recommendations. We expect significant benefits for patients, physicians and the public health system.

<u>Specific Aim 1:</u>

To evaluate the chance of a live birth within one year since the time of randomization between women allocated to surgery and those allocated to IVF.

<u>Specific Aim 2.1:</u>

To compare the cost-effectiveness of the two approaches using the health care system perspective.

<u>Specific Aim 2.2:</u>

To assess whether the systemic inflammatory milieu characteristic of endometriosis may have an impact on the IVF outcomes and on the quality of folliculogenesis and embryological development. through the analysis of steroid hormones in follicular fluid and circulating extracellular vesicles (EV)-immune and miRNA signatures. This aim will be pursued through the characterization and analysis of circulating extracellular vesicles (EV) immunologic, proteomic and miRNA signatures and measurement of steroid hormones in follicular fluid.

3. EXPERIMENTAL DESIGN

The study is a randomized controlled study comparing the chance of pregnancy between infertile women with sonographically identified endometriosis undergoing surgery and those treated with IVF.

The study is not blinded because it is not possible to carry out the treatments provided without the patient and the doctor becoming aware of it. The starting point will be the moment of randomization. Women who agree to participate in the study will be randomized to surgery followed by natural pregnancy research (arm 1) or to three complete cycles of IVF (i.e. three egg retrievals regardless of the number of embryo transfers) (arm 2). The patients, in both groups, will undergo the treatment in the shortest possible time, maximum 3 months. Only live births from pregnancies that began within 12 months of the start of randomization will be included in the primary results. The duration of 12 months was decided on the basis of data in the literature for which the majority of spontaneous pregnancies after surgery are expected within 9 to 12 months of surgery (Adamson and Pasta, 2010) and because the same period of time is necessary for carry out three complete cycles of IVF. Extending the period to two years would not provide any significant advantage for the recruited women, neither in the IVF arm nor in the surgery arm, as for both the chances of success are destined to progressively decrease.

As regards the secondary objectives, the economic analysis will be carried out using the data of the NHS applying the reimbursements of the Lombardy Region. The costs of hospital care (surgery, IVF procedures, treatment of any complications from surgery or IVF, obstetric care) will be calculated according to the regional reimbursements following the diagnosis groups. Drug costs will be included exclusively for drugs for which a reimbursement from the NHS is provided (mainly gonadotropins). Costs of infertility diagnosis will not be included as they are similar in the two arms. On the contrary, the additional costs necessary to cope with any complications from surgery or IVF and the costs necessary for the management of obstetric complications in the two arms (including neonatal care) will be included. In fact, differences in the course of pregnancies obtained by the patients placed in the two arms cannot be excluded. The main focus is on the cost of getting a live baby.

Finally, to achieve the secondary biological objectives, only women included in the IVF arm will be recruited for this part of the study. They will be matched, to a control group of women without

endometriosis, in a 1: 1 ratio based on ovarian reserve (± 0.2 ng / ml in AMH), age (± 1 year), starting dose of gonadotropins (same drug and identical dose) and study period. This part of the study will be carried out in two units (Coordinating Center and San Raffaele Hospital) with the aim of including the first 50 cases recruited and the related 50 controls. The women included will be subjected to:

- collection and isolation in peripheral blood of extracellular vesicles (IV) as the main mediators of the systemic inflammatory condition that can interfere with the results of IVF. These will be analyzed centrally at EPIGET Lab. EVs will be evaluated by: i) Nanoparticle Tracking analysis (NTA) to determine their total values and their distribution; ii) specific markers for the various lymphocyte populations by flow cytometry to evaluate the immunological origin; iii) miRNA profile and iv) proteomic analysis in a minor subset of samples (25 cases and 25 controls).

-collection of follicular fluids from egg retrieval and dosage of steroid hormones. Furthermore, the characteristics of the embryos will be related to the concentrations of steroid hormones in the follicular fluids. These will be analyzed centrally at UOS Environmental and Industrial Toxicology.

<u>Inclusion criteria are as follows</u>:

• Age < 40 years

• Pregnancy seeking for more than 12 months

• Regular menstrual cycle, i.e. mean cycle interval between 21 and 35 days

• Ultrasonographic diagnosis of ovarian endometriomas or deep peritoneal endometriosis.

Normal seminal analysis based on WHO criteria

• Absence of ureteral stenosis or intestinal subocclusive symptoms.

Even severe pain symptoms will not be exclusion criteria. Patients with pain symptoms destined for IVF will be treated for pain with hormone therapy (progestogen, estrogen-progestogen, or similar GnRH) until attempted. These therapies will be suspended only during the period necessary to carry out the IVF cycle. In case of non-resolution of painful symptoms, the patient will be referred for surgery but will be kept in the IVF arm ("intention to treat" analysis).

<u>Inclusion criteria for biological analysis controls</u>

<u>Exclusion criteria are as follows:</u>

• Previous surgery for endometriosis
• Previous IVF cycles
• Contraindication to pregnancy
• Hydrosalpinx
• Endometriomas with a mean diameter > 4 cm
• Submucosal fibroids or large intramural or subserosal fibroids (_ 5 cm).
• Doubtful sonographic findings that do not allow to reliably rule out malignancy.
• Obstacles to regular sexual intercourses (sexual disturbances or logistic problems)

Exclusion criteria for biological analysis controls

Egg retrieval for fertility preservation

Severe pain symptoms will not be an exclusion criterion. Women with these symptoms allocated to IVF will be firstly treated with proposed hormonal treatment (progestins, estroprogestins or GnRH analogues) to manage their pain prior to embark in IVF. These treatments will be transiently discontinued only during the IVF attempts. In case of pain-resistance to hormonal treatment, the woman will be scheduled to surgery but maintained in the IVF arm (intention to treat analysis).

The infertility work-up will include: semen analysis, serum assessment of AMH and a transvaginal assessment including also antral follicle count (AFC). The ultrasound assessment will be performed according to international recommendations (Guerriero *et al.*, 2016).

Women accepting to enter the study will be randomized to either surgery and then natural pregnancy seeking or a program of three complete IVF cycles (i.e. three oocytes retrievals regardless of the number of embryo transfers performed). The initial time point will be the time of randomization. Women of both study groups will initiate treatment (surgery or IVF) in a shortest delay, maximum 3 months. Only live birth pregnancies and initiating within a 12-months period starting from this time point will be included in the primary outcome. The duration of 12 months was decided because most natural pregnancies are expected to occur during the first 9-12 months after surgery (Adamson and Pasta, 2010) and this same duration of time is needed to perform three complete IVF cycles. Extending this duration longer (two years) would be useless
for women scheduled to IVF and could be unfair for women allocated to surgery (the chances of success with IVF could be significantly reduced).

After surgery, patients will be informed about their chances of natural pregnancy using the Endometriosis Fertility Index (EFI) questionnaire (Adamson and Pasta, 2010). Women unsatisfied with the prospected prognosis will be allowed to access IVF earlier.

Since both approaches (IVF and surgery) are supported by the public health system, the study will not cover the financial resources required for treatments. Both approaches will be performed according to local standards.
Sample size calculation is based on the following assumptions: 1) expected success rate in the surgical group: 30%, 2) type I and II errors of 0.05 and 0.20, 3) difference in favour of IVF justifying the additional costs of the procedure of 20% (absolute rate of success of 50%). On these bases, the number of women to be randomized is 206 (103 per arm). Considering an expected rate of eligible women declining treatment of about 30%, the total number of women to be initially selected would be 300.
The economic analyses will be performed using the public health system perspective by applying the reimbursements used in Lombardy. Costs of hospital care (surgery, and IVF procedures, management of IVF or surgical complications, obstetrics care) will be obtained using local reimbursements of diagnosis related groups (DRG). Costs of drugs will exclusively include those that are reimbursed by the public health system (mainly gonadotropins). Costs of infertility assessments will not be included since considered similar in the two arms of the study. In contrast, we will include additional costs needed to handle complications of surgery or IVF in the two arms as well as those required for obstetrics management of complications (including neonatal assistance). Indeed, we cannot exclude differences in pregnancy course between the deviations (leaving natural pregnancy seeking after surgery earlier or deciding for surgery prior
to complete the three IVF attempts or drop-out from IVF program without switching to surgery) will not justify exclusion. Somehow, even if less pure, evidence that will be obtained with our study will better stick to everyday clinical practice.
A main possible cause of reluctance to participate or deviation could be age and ovarian reserve testing. Indeed, there is an unproven tendency to over-estimate the detrimental effects of timing on the chances of IVF. Age and ovarian reserve are crucial for IVF success but the impact of a one-year delay is modest. For instance, in a recent theoretical model, we highlighted that even in women older than 35 years, a 6-months delay in the access to IVF is unremarkable (Somigliana *et al.*, 2020). Moreover, it is important to underline that the amount of ovarian reserve does not affect the chances of natural pregnancy (Somigliana *et al.*, 2015b) and one may speculate that surgery could be even wiser in women with reduced ovarian reserve. To note, a low ovarian reserve testing was shown to be poorly predictive of premature menopause (Depmann *et al.*, 2018). A specific seminar dedicated to the complex relation among age, ovarian reserve, natural pregnancy and IVF success

will be organized prior to initiate recruitment for all physicians who will be engaged in the study to prevent undue beliefs that could ultimately be deleterious for recruitment.

4. PROCEDURES

Interventions

Women accepting to enter the study will be randomized to either surgery and then natural pregnancy seeking or a program of three complete IVF cycles (i.e., three oocyte retrievals regardless of the subsequent number of embryo transfers that will be possible). Both approaches will be performed according to local standards. The initial time point will be the time of randomization. Eligible patients will undergo a first visit of screening. They will undergo a transvaginal ultrasound control, following international guidelines for the diagnosis of endometriosis (Guerriero et al, 2016). Women eligible for the study will be referred by care providers to a member of the research team who will describe the informed consent process. Details of the study will be explained to patients, including its main aims, procedures, temporal commitment, possible discomforts and risks, benefits. Recruitment will be on a voluntary basis with the right to withdraw from the study at any time; moreover, women will be informed that the decision to join the study protocol will not affect their possibility to shift to the other technique (surgery or IVF). Finally, informed consent will be illustrated and provided to the patients willing to participate. Operators will give patients the opportunity for questions and enough time to consider their participation. After two weeks, patients who will agree to enter the study will refer again to return the signed consent and to be randomized. During this visit, demographic and clinical characteristics as well as ultrasound findings will be recorded. Randomization will be organized centrally by Redcap (version for Fondazione IRCCS Ca Granda Ospedale Maggiore Policlinico, Milano : https://redcap.policlinico.mi.it/). The allocation sequence will be computer-generated. The allocation ratio will be 1:1. Randomization list will be stratified for the three participating centres. All patients, all caregivers and embryologists at the clinical departments will not be blinded to trial intervention allocation after inclusion. The progress of the study will be periodically monitored by an external monitor in order to verify the strictness of the data management.

Once randomized, both patients and physicians will not be blinded to the treatment arm. Women of both study groups will initiate treatment (surgery or IVF) in a shortest delay, maximum 3 months. After surgery, patients allocated in this arm, will be informed about their chances of natural

pregnancy using the Endometriosis Fertility Index (EFI) questionnaire (Adamson and Pasta, 2010). Patients allocated in IVF arm will undergo three complete IVF cycles (i.e. three oocyte retrievals regardless of the number of embryo transfers performed). Visits in both arms will be done every 3 months. Patients will be controlled about symptoms or new endometriotic lesions throughout ultrasound scans. Final visit will take place 12 months from randomization. If pregnant, women will be contacted even later to assess the evolution of pregnancy.

Even if patients will be informed and made aware about the importance of persisting in the allocated arm because inefficacy of treatment cannot be drawn prior to complete the whole course (surgery + 9-12 months of natural pregnancy seeking versus three completed IVF cycles), they will be allowed to switch to the alternative treatment at any time (pragmatic trial). However, for the analyses, they will remain in the originally allocated arm (intention to treat). A per protocol analysis will also be done, but this is not the primary intent. Reasons surrounding the decision to switch will be recorded. At the end of the study period (12 months), women failing to conceive will be counselled about the option to persist in the allocated arm versus performing the cross-over; i.e. women who will be allocated to surgery will be offered IVF and the other way around for those allocated to IVF.

Given the uncertainty regarding the willingness to participate to a RCT comparing two radically different approaches, we will propose to women refusing randomization to participate to a parallel study that will assess the same outcomes as those who will be randomized, even if the decision is taken by patients rather than being randomly sorted (*patient preference trial*).

For specific aim 3, only women scheduled for IVF will be included in this part of the study.Women included will be subjected to:

A. *collection and isolation of peripheral blood EVs* as mediators of systemic inflammatory conditions that may affect IVF outcomes. EVs will be evaluated for i) Nanoparticle tracking analysis (NTA) to determine total EV counts and size distributions; ii) Immunological origins targeting the immune system population markers by flow cytometry; iii) miRNA and iv) proteomic signatures.

B. *collection of the follicular fluid during oocyte retrievals* and assessment of the whole steroids cascade. Comparisons will be done between women with and without endometriosis. In addition, embryological variables will be recorded and correlated with steroid hormone concentrations.

Peripheral blood will be collected prior to initiate the IVF cycle and the plasma fraction will be used to purify EVs. Plasma will be centrifuged three times at 1000, 2000 and 3000 × *g* for 15 min at 4°C,

and the pellet will be discarded to clean the cell debris. The vesicles will be then concentrated by ultracentrifugation at 110,000 × *g* for 120 min at 4°C. In order to obtain intact EVs, samples cannot be frozen and specimens need to be processed within 3 days from blood drawing. This is the main reason for recruiting new subjects instead of starting from biobanked samples.

NTA will be used to determine total EV counts and size distributions. EVs will be characterized for immune signatures using the MACSQuant Analyzer flow cytometer (Miltenyi Biotec, USA). EV analysis by flow cytometry is quite challenging for the limited EV size. To evaluate EV integrity, sample aliquots will be stained with carboxyfluorescein diacetate N-succinimidyl ester (CFSE) at 37°C for 20 min in the dark. CFSE is a vital non-fluorescent molecule that can enter EVs, where intracellular esterases remove the acetate group and convert the molecule into the fluorescent ester form. Each aliquot of CFSE-stained sample will then be incubated with specific antibodies to investigate the possible cellular origin of EVs: Ab-CD14$^+$ (monocytes/macrophages), Ab-CD127$^+$/ Ab-CD25$^+$ (Tregs), Ab-CD4$^+$/Ab-CXCR3$^+$ (Th1 cells), Ab-CD4$^+$/Ab-CCR4$^+$ (Th2 cells). Quantitative multiparameter analysis of flow cytometry data will be performed using FlowJo software (Tree Star, Inc., Ashland, OR, USA). Following extraction of EV-miRNAs using the commercial miRNeasy Mini and RNeasy MiniElute kits (Qiagen, Frederick, MD, USA), miRNAome (754 miRNAs) will be analyzed by QuantStudio™ 12K Flex Real-Time PCR System (Thermo Fisher). miRNAs associated most closely with inflammation-related endometriosis will be further confirmed by real time PCR in the rest of the samples.

Regarding proteomic signature, EVs will be lysed, proteins will be quantified and digested. Samples will be injected in duplicate on an nLC–ESI–MSMS quadrupole Orbitrap QExactive-HF mass spectrometer (Thermo Fisher Scientific). Peptides will be separated on UHPLC Easy-nLC 1000 (Thermo Fisher Scientific) connected to a 25 cm fused-silica emitter of 75 µm inner diameter (New Objective, Inc.), packed in-house with ReproSil-Pur C18-AQ 1.9 µm beads. MS data will be acquired using a data-dependent top 15 method for HCD fragmentation. Survey full scan MS spectra (300–1650 Th) will be acquired in the Orbitrap with 60 000 resolution, AGC target 3e6, IT 20 ms. For HCD spectra, resolution will be set to 15 000 at m/z 200, AGC target 1e5, IT 80 ms; NCE 28% and isolation width 1.2 m/z. For quantitative proteomics, raw MS files will be processed with MaxQuant (version 1.5.2.8) using label-free quantification, searching against the database Uniprot_cp_human_setting. The peptides and protein FDR will be set to 0.01; the minimal length required for a peptide will be six amino acids, and a minimum of two Label-free quantification (LFQ) intensity will be imported into Perseus (version 1.5.0.31) applying imputation and Z-score

transformation. Statistical analysis will be performed using t-test, Benjamini–Hochberg correction, FDR 0.05.

5. ENDPOINT

Primary endpoint:

to assess whether IVF is more effective than surgery in obtaining a live birth and, if so, what is the magnitude of this benefit.

Secondary endpoint:

to assess whether or not IVF is more cost-effective than surgery; to understand whether the endometriosis-related systemic inflammatory milieu demonstratedcby the presence of circulating EVs characterized by an inflammatory signature may influence the folliculogenesis quality and IVF outcomes.

6. STUDY DURATION

The study will last three years. Recruitment will terminate within one year 20 months after first patient's inclusion. The study will initiate once obtained the acceptance of the ethical committee and study registration done. Thereafter, patients will be followed up until the end of the study period. The last few months will be dedicated to analyses and drafting of the papers.

0 to 3 months: Ethical committee, personnel identification, tutorial activities

4 to 15 months: Recruitment

16 to 36 months: Follow-up, data entry, biological analyses, data analyses and papers drafting

16 to 48 months: Follow-up cross-over patients, data analyses and papers drafting

7. STATISTICAL ANALYSIS

Sample size

Sample size calculation is based on the following assumptions: 1) expected success rate in the surgical group: 30%, 2) type I and II errors of 0.05 and 0.20, 3) difference in favour of IVF justifying the additional costs of the procedure of 20% (absolute rate of success of 50%). On these bases, the number of women to be randomized is 206 (103 per arm). Considering an expected rate

of eligible women declining treatment of about 30%, the total number of women to be initially selected would be 300.

8. ADVERSE EVENTS

The project does not envisage the administration of drugs or other substances or clinical practices outside the standard of care usually proposed to these patients. The adverse events that may occur are therefore those related to the carrying out of surgical therapy or the IVF cycle and will not differ from what is expected in normal clinical practice.

9. RISK/BENEFIT ASSESSMENT

The study does not involve additional risks for the patients as they are patients who, due to the presence of endometriosis, would in any case be subjected to one of the two treatments. Specifically, the risks associated with the IVF procedure can be viewed in the Consent in use at UOSD PMA M.01.732.cons and those connected with surgical treatment in the Consent in use at the Gynecology Unit.

The study will instead have immediate benefits for the national health system (NHS). Considering that the goal is to identify the best option for infertile women with endometriosis both clinically and economically, the results could be used by the NHS to refine local guidelines for the management of endometriosis and to stem the large heterogeneity. of treatments currently in place on the national territory. More importantly, it will be possible to advise women on the basis of robust data rather than theoretical considerations derived from weak evidence.

Furthermore, we expect valuable insights from the investigation into the harmful effects mediated by inflammation of endometriosis. First, this biological evidence could better elucidate the mechanisms of infertility related to endometriosis. Second, it could allow us to identify possible targets for the development of new therapeutic agents. Finally, new biomarkers of impaired infertility could emerge, thus allowing women to be selected correctly.

10. STUDY MANAGEMENT

<u>Data collection and management</u>

Each participant, at the time of enrollment, will be assigned a unique code. The file that associates the participant's code with the relevant identification data will be stored separately on a password-protected computer. The study database will be password protected and uploaded to a computer which is also password protected and accessible only to study personnel designated by the principal investigator. The de-identification of the data will take place in such a way that the people who access the database will not be able to trace the identity of the subjects in any way. Only local investigators will be able to trace the identity of the subjects enrolled.

Data collection will be centralized online through the CRF of the Polyclinic. The data necessary for the study will be recorded in a specific eCRF in a Data Management System validated according to national legislation, provided by the Scientific Direction of the Foundation. The platform used will be RedCap (Research Electronic Data Capture).

The REDCap Consortium is made up of 1000 institutional partners around the world (research bodies, universities, ministries, etc.). The consortium supports a secure web application (REDCap) designed solely to support data acquisition for research studies. The REDCap application allows users to create and manage databases online quickly and securely, and is currently in use for more than 110,000 projects with approximately 150,000 users covering numerous research areas of interest across the consortium.

Through REDCap, for this study will be implemented: a) identification at user level, with specific restrictions based on the role in the study b) validation and control of data integrity in real time c) identification of patients before data export d) centralized data storage with daily backup, a secure server within the IT structure of the Foundation.

Approval by the Ethics Committee

The investigator will ensure that the protocol has been viewed and approved by the local Independent Ethics Committee (EC) before starting the study. The CE will also need to verify and approve the Informed Consent (IC) form and all written information received from the patient prior to enrollment in the study.

In case of the protocol or IC edits during the study, the investigator will be the guarantor and therefore the person in charge of ensuring the review and approval of this modified document as requested by the EC. The content of these changes will only be implemented after the CE has

approved them. Until then, it will be necessary to refer to the previous version of the document already approved.

Informed Consent

The investigator or other staff appointed by him is responsible for informing people about all aspects and procedures of the study. The process for obtaining informed consent must comply with the regulatory procedures in force. The investigator (or designated associate) and subject must date and sign the informed consent form before the patient initiates any study-related procedures. The subject will receive a copy of the CI dated and signed by both. The original copy will be kept in the archives designated for the study. Neither the investigator nor designated personnel should in any way exert any coercion or influence on a subject to induce him to participate or continue to participate in the study. A subject's decision to participate in the study must be completely voluntary. The investigator and designated personnel must emphasize to the individual that they can withdraw their consent at any time without penalty or loss of any benefit to which they may be entitled.

The written or oral information relating to the study, including the written consent form, does not contain any linguistic expressions that compel the subject to waive (even apparently) his legal rights, or that exempt the investigator, institution or sponsor from liability for negligence.

Duties of the investigator

In accordance with applicable local regulations, the investigator will send periodic reports regarding the progress of the study in his center to the EC and notify the EC of the closure of the study. Periodic reporting and termination notification are part of the investigator's responsibilities.

Study monitoring

In accordance with applicable regulations and good clinical practice (GCP), the monitor will periodically visit or contact the center. The duration, nature and frequency of these visits / contacts depend on the frequency of recruitment, the quality of the documents held by the center and their adherence to the protocol. They will be decided with the monitor.

Through these contacts, the monitor must:

- check and evaluate the progress of the study

-examine the data collected

-conduct the verification of the source document,

- identify each problem and related solutions.

The purposes of the monitoring activity are to verify that:

-the rights and welfare of the subject are respected

-the study data are accurate

-complete and verifiable from the original documents

-the study is conducted in accordance with the protocol and any approved amendments, GCP and applicable regulations

The investigator must:

-give the monitor direct access to all relevant documentation

-devote some of his or her staff and time to the monitor to discuss the results of the monitoring and any other possible aspects.

The monitor will contact the center prior to the start of the study to discuss the protocol and data collection procedures with the staff. In consideration of the length of the follow up (up to two years) and the recruitment period (12 months), no interim analyzes are foreseen (almost all the patients recruited will still be in progress at the time of the end of the recruitment).

Any decisions on the interruption or modification of the trial will be made in agreement between the PI and all the participants previously listed. In case of discrepancy we will proceed by vote, in case of a tie the PI's decision will prevail.

Since the study is funded by the Ministry of Health (Finalized research grant), no significant conflicts of interest are expected. However, the personnel involved in the study inevitably have personal conflicts of interest that will be collected and declared at the time of the drafting of the study.

The monitoring will be managed by an independent external monitor.

Study quality assurance

As promoter, Fondazione IRCCS Ca 'Granda, Ospedale Maggiore Policlinico can carry out a quality check on the study at its discretion. In this case, the investigator will allow the monitor to directly access all relevant documentation and devote some of his or her time and staff to the reviewer to discuss the results of the monitoring and any other aspects of the study.

Furthermore, the Regulatory Authorities will be able to carry out inspections. In this case, the investigator will give the inspector direct access to all relevant documentation, and will devote some of his or her time and staff to the inspector to discuss the results of the monitoring and any other aspects of the study.

Closure of the study

Upon closure of the study, the monitor and investigator will activate a series of procedures: review all study documentation, reconcile study data, reconcile all clarification reports.

Archiving of documents

In compliance with current national regulations, the investigator will keep a copy of all documentation in a dry and safe place after the closure of the study (For the Coordinator Center: Room 15, PMA Regina Elena Center).

Disclosure of information regarding scientific findings

The investigator and other personnel involved in the study will treat all information relating to the study (including the protocol, data obtained and all documentation produced during the study) confidentially and will not use such information, data or reports for purposes other than those described in the protocol.

These restrictions do not apply to: 1) information that becomes publicly available, not through negligence on the part of the investigator or his staff; 2) information that requires confidential disclosure to CE for the sole purpose of evaluating the study; 3) information that must be disclosed in order to obtain adequate medical care for a study subject.

Publication

The scientific managers of the study will undertake to draft a final report and to make the results public at the end of the study. The data will be made public anonymously and presented as required in aggregate mode (however, the data may be sent to the scientific journal in non-aggregated and de-identified form, if specifically requested). The results will be disseminated through communications at national and international conferences and publications in scientific journals following the international guidelines for defining authorship. In the case of separate publications, each publication must mention that these results have been achieved in the context of this Project.

Intellectual property rights of the study results

The Promoter of the Study and the Participating Centers mutually acknowledge that during the implementation of the Project, within the limits of what is strictly necessary to carry it out, know-how, technical material and / or protected assets could be used to varying degrees industrial and / or intellectual property rights or those susceptible to protection, developed before the start of the Project by the Promoter and by the Participating Centers which remain the owners.

The results of the research activities will remain the property of the Promoter and the Participating Centers in proportion to the respective inventive contribution and their use, it being understood that the provision of biological samples at the basis of the research project, related clinical information and related medical know-how by the study promoter will be considered as an essential contribution.

In the event of innovative results, susceptible to patent protection (or similar patent right) and / or economic exploitation, the joint owners undertake to regulate in a specific agreement the methods of protection and enhancement of said results. In this case, any publications will be subject to the completion of all procedures for the patent protection of the results.

11. COMPENSATION IN THE EVENT OF DAMAGES

In the event of undesirable events or any damage that may derive from participation in the research, the Insurance Policy of our Institute is also extended to cover the subjects participating in the research projects.

12. AMENDMENTS TO THE PROTOCOL

Any changes to the protocol will be promptly requested from the competent Ethics Committee.

13. FINANCIAL ARRANGEMENTS

This project was funded by the Ministry of Health - Finalized Research Call 2019.

14. DISCLOSURE (attached)

15. REFERENCES

Adamson GD, Pasta DJ. Endometriosis fertility index: the new, validated endometriosis staging system. Fertil Steril. 2010 Oct;94(5):1609-15.

Bafort C, Beebeejaun Y, Tomassetti C, Bosteels J, Duffy JM. Laparoscopic surgery for endometriosis. Cochrane Database Syst Rev. 2020 Oct 23;10:CD011031.

Duffy JMN. COMMIT: Developing a core outcome set for infertility research. Hum Reprod. Abstract book 2019; 34 Suppl. 1, pp 171, O-218.
Accessible at: *https://cm.eshre.eu/presentations/ESHRE2019/O-219/default.aspx*

ETIC Endometriosis Treatment Italian Club. When more is not better: 10 'don'ts' in endometriosis management. An ETIC position statement. Hum Reprod Open. 2019 Jun 12;2019(3):hoz009.
Guerriero S, Condous G, van den Bosch T, Valentin L, Leone FP, Van Schoubroeck D, Exacoustos C, Installé AJ, Martins WP, Abrao MS, Hudelist G, Bazot M, Alcazar JL, Gonçalves MO, Pascual MA, Ajossa S, Savelli L, Dunham R, Reid S, Menakaya U, Bourne T, Ferrero S, Leon M, Bignardi T, Holland T, Jurkovic D, Benacerraf B, Osuga Y, Somigliana E, Timmerman D. Systematic approach to sonographic evaluation of the pelvis in women with suspected endometriosis, including terms, definitions and measurements: a consensus opinion from the International Deep Endometriosis Analysis (IDEA) group. Ultrasound Obstet Gynecol. 2016 Sep;48(3):318-32.

Harb HM, Gallos ID, Chu J, Harb M, Coomarasamy A. The effect of endometriosis on in vitro fertilisation outcome: a systematic review and meta-analysis. BJOG. 2013 Oct;120(11):1308-20.

Hodgson RM, Lee HL, Wang R, Mol BW, Johnson N. Interventions for endometriosis-related infertility: a systematic review and network meta-analysis. Fertil Steril. 2020 Feb;113(2):374-382.e2.

Nisenblat V, Bossuyt PM, Farquhar C, Johnson N, Hull ML. Imaging modalities for the noninvasive diagnosis of endometriosis. Cochrane Database Syst Rev. 2016 Feb 26;2(2):CD009591.

Somigliana E, Benaglia L, Paffoni A, Busnelli A, Vigano P, Vercellini P. Risks of conservative management in women with ovarian endometriomas undergoing IVF. Hum Reprod Update. 2015 Jul-Aug;21(4):486-99

Somigliana E, Vigano P, Benaglia L, Busnelli A, Berlanda N, Vercellini P. Management of Endometriosis in the Infertile Patient. Semin Reprod Med. 2017 Jan;35(1):31-37.

Somigliana E, Viganò P, Benaglia L, Busnelli A, Paffoni A, Vercellini P. Ovarian stimulation and endometriosis progression or recurrence: a systematic review. Reprod Biomed Online. 2019 Feb;38(2):185-194.

Vercellini P, Somigliana E, Viganò P, Abbiati A, Barbara G, Crosignani PG. Surgery for endometriosis-associated infertility: a pragmatic approach. Hum Reprod. 2009 Feb;24(2):254-69.

Vercellini P, Barbara G, Buggio L, Frattaruolo MP, Somigliana E, Fedele L. Effect of patient selection on estimate of reproductive success after surgery for rectovaginal endometriosis: literature review. Reprod Biomed Online. 2012 Apr;24(4):389-95.

Vercellini P, Viganò P, Somigliana E, Fedele L. Endometriosis: pathogenesis and treatment. Nat Rev Endocrinol. 2014 May;10(5):261-75.

Vercellini P, Viganò P, Frattaruolo MP, Borghi A, Somigliana E. Bowel surgery as a fertilityenhancing procedure in patients with colorectal endometriosis: methodological, pathogenic and ethical issues. Hum Reprod. 2018 Jul 1;33(7):1205-1211.

WHO laboratory manual for the examination and processing of human semen Fifth edition, 2010 Available at:*https://www.who.int/reproductivehealth/publications/infertility/9789241547789/en/*
