# Supplementary material for: Surgery versus IVF for the treatment of infertility associated to ovarian and deep endometriosis (SVIDOE: Surgery Versus IVF for Deep and Ovarian Endometriosis). Clinical protocol for a multicenter randomized controlled trial
Source: PLoS One. 2022 Aug 3;17(8):e0271173. doi: 10.1371/journal.pone.0271173 (PMC9348732; doi:10.1371/journal.pone.0271173)
Supplement: S2 Protocol — (DOCX) [file pone.0271173.s003.docx]

**FIVET versus chirurgia per il trattamento dell’infertilità associata a endometriosi ovarica e profonda.**

Acronimo: SVIDOE (Surgery Versus IVF for Deep and Ovarian Endometriosis)

Numero della versione del protocollo: v.1.0 Data: 11/12/2020

Promotore: Fondazione IRCCS Ca’ Granda Ospedale Maggiore Policlinico, Via Sforza 28, 20122 Milano, Italia

Centro coordinatore: U.O.S.D. Procreazione Medicalmente Assistita Fondazione IRCCS Ca’ Granda Ospedale Maggiore Policlinico, Via M. Fanti 6, 20122 Milano, Italia

Sperimentatore Principale: Laura Benaglia

PI Centro Satellite OSR: Jessica Ottolina

FLOWCHART
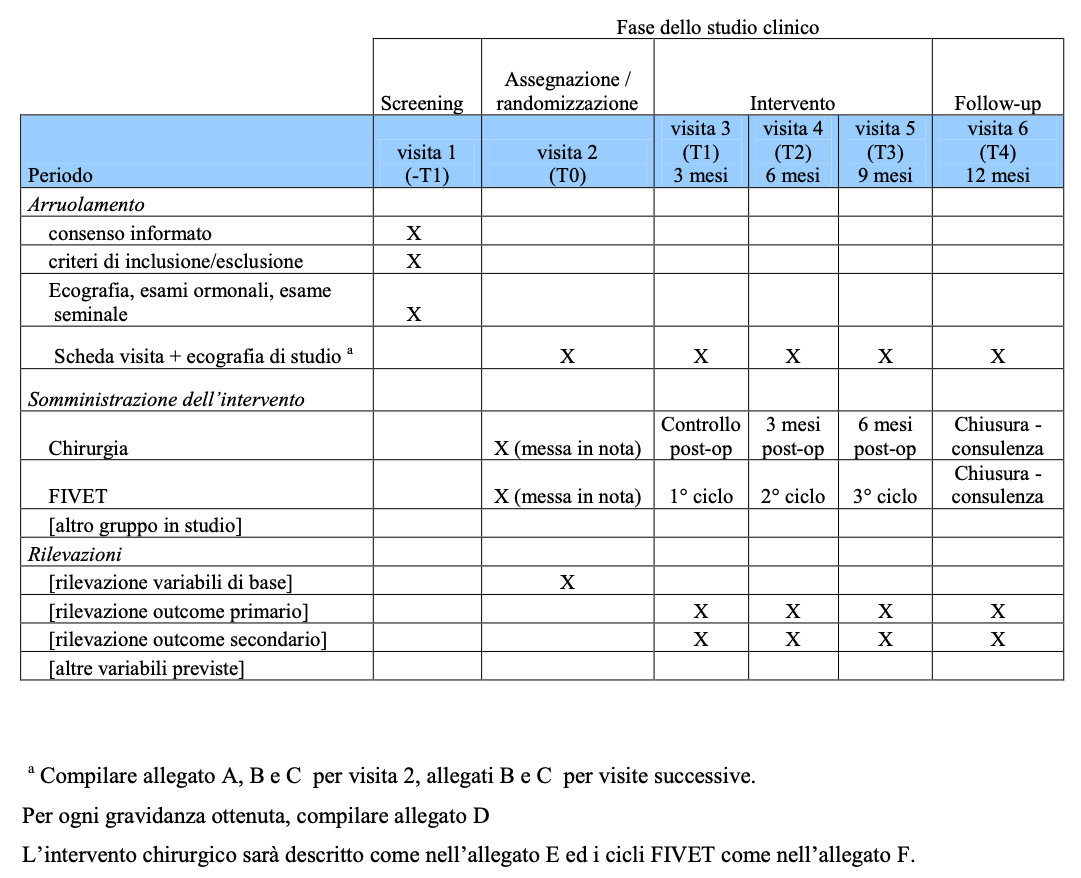


LISTA DELLE ABBREVIAZIONI

95%IC: Intervallo di confidenza al 95%
AMH: Anti-mullerian hormone (ormone anti-mulleriano)
CE: Comitato Etico
CI: Consenso Informato
CRF: Case Report Form, scheda raccolta dati
DRG: Gruppi di diagnosi (diagnosis related groups)
GCP: Good Clinica Practice, buona pratica clinica
FIVET: Fecondazione in Vitro e Embryo Transfer
NNT: Number needed to be treated
NTA: Neuroparticle Tracking Analysis
OR: Odds Ratio
PMA: Procreazione medicalmente assistita
SSN: Sistema Sanitario Nazionale
UO : Unità operativa
UOC: Unità Operativa Complessa
UOS: Unità operativa Semplice
UOSD: Unità Operativa Semplice Dipartimentale
WHO: World Health Organization (Organizzazione Mondiale della Sanità)

RESPONSABILITA’ (ruolo del promotore e dei collaboratori)

Collaborazioni interne

La Fondazione IRCCS Ca' Granda, Ospedale Maggiore Policlinico è promotore dello studio. La UO1 è formata dalla UOSD PMA (Direttore Prof. Edgardo Somigliana) e la UOC Ginecologia (Direttore Prof. Paolo Vercellini)
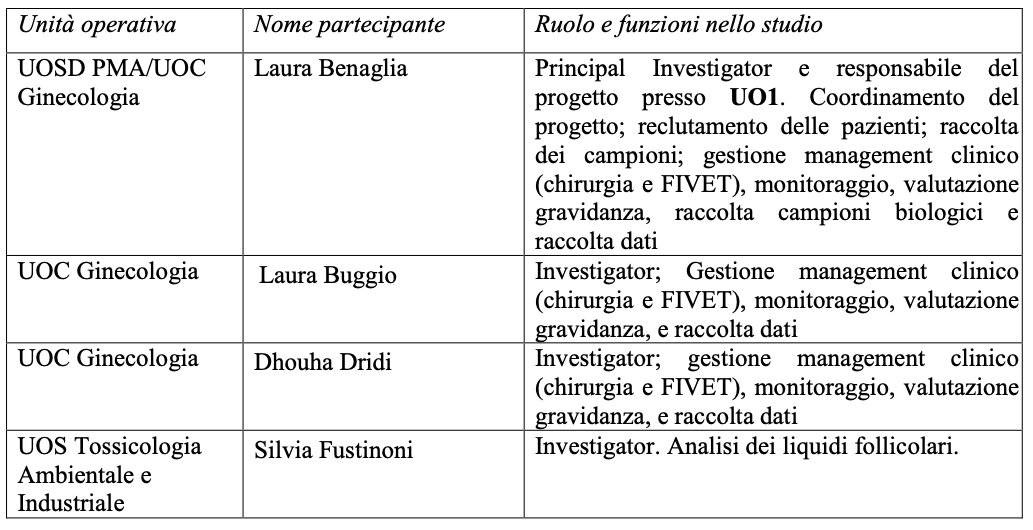


Collaborazioni esterne (analisi campioni biologici, analisi dati, procedure diagnostiche, etc.)
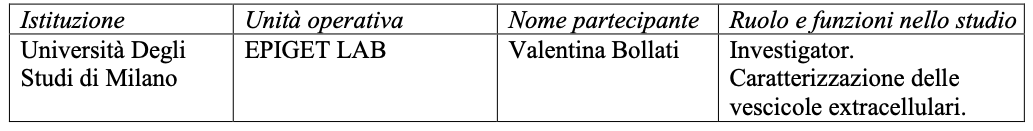


INDICE

1. INTRODUZIONE

1.1 Background e razionale

2. OBIETTIVO DELLA SPERIMENTAZIONE

2.1 Obiettivo primario

2.2 Obiettivo/i secondario/i

3. DISEGNO DELLO STUDIO

3.1 Disegno dello studio 3.2 Criteri d’inclusione 3.3 Criteri di esclusione

4. PROCEDURE RELATIVE ALLO STUDIO

4.1 Intervento
4.2 Randomizzazione

4.3 Cecità

5. ENDPOINT

5.1 Endpoint primario

5.2 Endpoint secondario

6. DURATA/TIMELINE DELLO STUDIO

7. ANALISI STATISTICA

7.1 Dimensione del campione

7.2 Analisi dei dati

8. EVENTI AVVERSI
9. VALUTAZIONE RISCHIO / BENEFICIO 10. GESTIONE DELLO STUDIO

10. GESTIONE DELLO STUDIO

10.1 Raccolta e gestione dei dati
10.2 Aspetti regolatori e considerazioni etiche

10.2.1 Approvazione dell’Autorità competente

10.2.2 Approvazione del Comitato Etico

10.2.3 Consenso informato
10.3 Doveri dello sperimentatore
10.4 Monitoraggio dello studio
10.5 Assicurazione della qualità dello studio
10.6 Chiusura dello studio
10.7 Archiviazione di documenti
10.8 Divulgazione di informazioni riguardanti la scoperta scientifica

10.8.1 Confidenzialità

10.8.2 Pubblicazioni
10.9 Diritti di proprietà intellettuale sui risultati dello studio

11. INDENNITA’ E RISARCIMENTO IN CASO DI DANNI

12. EMENDAMENTI AL PROTOCOLLO
13. ACCORDI FINANZIARI
14. DISCLOSURE SUI CONFLITTI DI INTERESSE

15. REFERENZE

16. ALLEGATI

1.1 BACKGROUND E RAZIONALE

Il trattamento delle pazienti con infertilità correlata all’endometriosi rimane tuttora controverso (Vercellini et al., 2014; Somigliana et al., 2017; Hodgson et al., 2020). I dati in letteratura sono solidi solo in merito all’endometriosi peritoneale superficiale, che è una condizione clinica la cui diagnosi necessita però un’esplorazione chirurgica. Secondo una recente meta-analisi Cochrane, l’odds ratio (OR) di ottenere una gravidanza evolutiva in utero in donne sottoposte a laparoscopia operativa per il trattamento di lesioni endometriosiche superficiali rispetto a una mera laparoscopia diagnostica è 1.89 (95%CI: 1.25-2.86) (Bafort et al., 2020). Tuttavia, è opinabile effettuare laparoscopie diagnostiche in tutte le donne che presentano infertilità inspiegata allo scopo di identificare e trattare quella parte di pazienti con endometriosi al primo stadio. Infatti, l’incremento assoluto delle probabilità di gravidanza è relativamente basso (8-10%) e la malattia è presente solo nel 30-50% delle donne (almeno la metà delle pazienti sarebbero quindi inutilmente esposte ai rischi della chirurgia). Il number needed to be treated (NNT), vale a dire il numero di donne che è necessario trattare per avere una singola gravidanza in più è troppo alto (ETIC, 2019).

Solide evidenze per le altre forme di endometriosi sono invece carenti. Ad oggi, non esistono né studi randomizzati controllati né studi prospettici comparativi atti a chiarire il potenziale beneficio della chirurgia rispetto alla procreazione medicalmente assistita (PMA) in donne con endometriosi ovarica o peritoneale profonda. Per queste forme di malattia, la situazione clinica è peraltro molto diversa perché, al contrario dell’endometriosi superficiale, la diagnosi può essere ottenuta in mondo non invasivo. Secondo una recente meta-analisi Cochrane, la sensibilità e la specificità dell’ecografia transvaginale nella diagnosi di cisti ovariche endometriosiche sono 0.93 (95%IC: 0.87-0.99) e 0.96 (95%IC: 0.92-0.99), rispettivamente (Nisenblat et al., 2016). Per l’endometriosi profonda, sensibilità e specificità sono 0.79 (95%IC: 0.69-0.89) e 0.94 (95%CI: 0.88-1.00), rispettivamente (Nisenblat et al., 2016). Inoltre, le modalità dell’esecuzione dell’ecografia sono oggi standardizzate e di patrimonio comune per i moderni ginecologici (Guerriero et al., 2016). Di fatto, la chirurgia oggi non è più indispensabile per la diagnosi di queste forme di malattia.

La possibilità di identificare forme di endometriosi senza dover percorrere la via chirurgica ha condotto a un cambiamento nella gestione clinica. Nello specifico, donne affette da infertilità associata a endometriomi ovarici o endometriosi peritoneale profonda hanno quindi già una diagnosi definitiva e possono scegliere tra due percorsi terapeutici: la chirurgia o la fecondazione in vitro (FIVET). La chirurgia, da un lato, ha l’intento di aumentare le probabilità di concepimento naturale. Tuttavia, il reale beneficio in termini di gravidanza è modesto (circa il 25-30%) (Vercellini et al., 2009; Vercellini et al., 2012; Vercellini et al., 2018) e le donne sono esposte ai rischi chirurgici, tra i quali il più temuto è il possibile danno ovarico post-chirurgico (Somigliana et al., 2015). La FIVET, dall’altro canto, sembrerebbe più efficace in termini di gravidanze ottenute (50- 60%) (Smith et al., 2015). Tuttavia, l’efficacia è minore rispetto ad altre indicazioni. Il Rischio relativo di gravidanza clinica in donne con endometriosi III-IV stadio è infatti pari a 0.79 (95%CI: 0.69-0.91) (Harb et al., 2013). Inoltre, la procedura è più costosa rispetto alla chirurgia ed è anch’essa non scevra di rischi. Infatti, le pazienti con endometriosi non operata che si sottopongono a FIVET sono esposte a rischi aggiuntivi rispetto a pazienti che la eseguono per altre indicazioni. In particolare, è stato osservato un aumentato rischio infettivo nelle pazienti con endometriomi e un aumentato rischio di progressione delle lesioni o di emoperitoneo spontaneo in gravidanza nelle donne con endometriosi profonda (Somigliana et al., 2015; Somigliana et al., 2019).

In questa situazione di incertezza e di complessa valutazione di rischi e benefici, la decisione tra i due trattamenti deve essere presa in condivisione con la paziente dopo approfondito counseling. La scelta deve anche tenere conto di alcune importanti variabili quali la storia chirurgica pregressa, eventuali sintomi algici, il beneficio tratto da terapie mediche, l’età, la riserva ovarica e la qualità del seme del partner. Tuttavia, la mancanza di studi randomizzati controllati non consente di fornire alle pazienti informazioni complete e precise. In questo panorama, non sorprende quindi l’estrema eterogeneità nella gestione clinica che si osserva oggi nel paese (si va da centri che eseguono sempre la FIVET a centri che eseguono sempre la chirurgia).

2. OBIETTIVO/I O IPOTESI DELLA SPERIMENTAZIONE

Lo scopo di questo studio è valutare la probabilità di nati vivi da gravidanze iniziate entro 1 anno dal reclutamento tra donne sottoposte a FIVET e donne sottoposte a chirurgia al fine di ottenere dati solidi che possano essere utilizzati nella pratica clinica quotidiana. Inoltre, valuteremo se l'ambiente infiammatorio caratteristico dell'endometriosi possa avere un impatto sugli esiti della FIVET, sulla qualità della follicologenesi e sullo sviluppo embrionario.

2.1. Obiettivo primario

Valutare la probabilità di nati vivi da gravidanze iniziate entro 1 anno dal reclutamento tra donne sottoposte a FIVET e donne sottoposte a chirurgia.

2.2. Obiettivo/i secondario/i
Gli obiettivi secondari dello studio sono di seguito riportati:

1. Economico: confrontare il costo-efficacia dei due trattamenti dal punto di vista della spesa sanitaria del SSN.
2. Biologico: valutare se il quadro infiammatorio cronico tipico dell’endometriosi possa avere un impatto sui risultati della FIVET e, dal punto di vista biologico, sulla follicologenesi e sullo sviluppo degli embrioni. Questo scopo sarà perseguito tramite l’isolamento e l’analisi del profilo di miRNA, immunologico e proteomico delle vescicole extracellulari circolanti e il dosaggio degli ormoni steroidei nel fluido follicolare.

3. DISEGNO DELLO STUDIO

3.1 Disegno dello studio

Lo studio è interventistico biologico non farmacologico multicentrico randomizzato controllato e confronta le probabilità di gravidanza in due gruppi di donne con diagnosi ecografica di endometriosi sottoposte a chirurgia o a FIVET. Lo studio non è in cieco perché non è possibile effettuare i trattamenti previsti senza che la paziente ed il medico ne vengano a conoscenza. Il punto di inizio sarà il momento della randomizzazione. Le donne che accettano di partecipare allo studio verranno randomizzate a chirurgia seguita da ricerca naturale di gravidanza (braccio 1) oppure a tre cicli completi di FIVET (ovvero tre prelievi ovocitari a prescindere dal numero di embryo transfer) (braccio 2). Le pazienti, in entrambi i gruppi, saranno sottoposte al trattamento nel minor tempo possibile, massimo 3 mesi. Solo i nati vivi da gravidanze iniziate entro 12 mesi dall’inizio della randomizzazione saranno inclusi nei risultati primari. La durata di 12 mesi è stata decisa sulla base di dati in letteratura per cui la maggior parte delle gravidanze spontanee dopo chirurgia è attesa entro 9-12 mesi dall’intervento (Adamson and Pasta, 2010) e perché lo stesso periodo di tempo è necessario per svolgere tre cicli completi di FIVET. Allungare il periodo a due anni non fornirebbe alcun significativo vantaggio per le donne reclutate, né nel braccio FIVET, né nel braccio chirurgia, in quanto per entrambe le probabilità di successo sono destinate a ridursi progressivamente.

Per quanto riguarda gli obiettivi secondari l’analisi economica sarà svolta utilizzando i dati del SSN applicando i rimborsi di Regione Lombardia. I costi delle cure ospedaliere (chirurgia, procedure FIVET, trattamento di eventuali complicanze da chirurgia o da FIVET, cure ostetriche) saranno calcolati secondo i rimborsi regionali seguendo i gruppi di diagnosi (DRG). I costi dei farmaci saranno inclusi esclusivamente per i farmaci per cui è previsto un rimborso dal SSN (prevalentemente gonadotropine). I costi della diagnosi di infertilità non saranno inclusi in quanto simili nei due bracci. Al contrario, saranno inclusi i costi addizionali necessari a fronteggiare eventuali complicanze da chirurgia o da FIVET e i costi necessari alla gestione di complicanze ostetriche nei due bracci (inclusa l’assistenza neonatale). Infatti, non si possono escludere differenze nell’andamento delle gravidanze ottenute dalle pazienti collocate nei due bracci. L’obiettivo principale è il costo per ottenere un bimbo nato vivo.

Infine, per raggiungere gli obiettivi secondari di tipo biologico, saranno reclutate per questa parte dello studio esclusivamente le donne incluse nel braccio della FIVET. Saranno abbinate, a un gruppo controllo di donne senza endometriosi, in rapporto 1:1 in base a riserva ovarica (± 0.2 ng/ml in AMH), età (± 1 anno), dose iniziale di gonadotropine (stesso farmaco e identica dose) e periodo di studio. Questa parte dello studio sarà svolta in due unità (Centro Coordinatore e Ospedale San Raffaele) con lo scopo di includere i primi 50 casi reclutati e i relativi 50 controlli. Le donne incluse saranno sottoposte a:

- raccolta e isolamento nel sangue periferico di vescicole extracellulari (EV) quali mediatori principali della condizione infiammatoria sistemica in grado di interferire con i risultati della FIVET. Questi verranno analizzati in modo centralizzato presso EPIGET Lab. Le EV saranno valutate mediante: i) Nanoparticle Tracking analysis (NTA) per determinarne i valori totali e la loro distribuzione; ii) marcatori specifici per le varie popolazioni linfocitarie tramite citofluorimetria a flusso per valutare l’origine immunologica; iii) profilo miRNA e iv) analisi proteomico in un sottogruppo minore di campioni (25 casi e 25 controlli).
- raccolta di fluidi follicolari da prelievi ovocitari e dosaggio di ormoni steroidei. Inoltre, le caratteristiche degli embrioni saranno correlate alle concentrazioni degli ormoni steroidei nei fluidi follicolari. Questi verranno analizzati in modo centralizzato presso UOS Tossicologia Ambientale e Industriale.

3.2. Criteri d’inclusione delle pazienti affette da endometriosi

Saranno inclusi pazienti che soddisfano contemporaneamente i seguenti criteri:

- pazienti che hanno firmato il consenso informato
- Età < 40 anni
- Ricerca prole da un tempo > 12 mesi
- Cicli mestruali regolari (ciclo medio 21-35 giorni)
- Diagnosi ecografica di endometriosi ovarica e peritoneale profonda.
- Esame del liquido seminale nella norma secondo i criteri WHO
- Assenza di stenosi ureterale o sintomi sub-occlusivi intestinali

3.3. Criteri di esclusione delle pazienti affette da endometriosi
Saranno esclusi pazienti che possiedono almeno uno dei seguenti criteri:

- Pregressa chirurgia per endometriosi
- Precedenti cicli FIVET
- Controindicazioni alla gravidanza
- Idrosalpinge
- Cisti endometriosica con un diametro medio > 4 cm
- Miomi sottomucosi (qualunque diametro) o voluminosi miomi intramurali o sottosierosi (≥ 5 cm)
- Riscontro ecografico dubbio per cisti ovarica maligna
- Impossibilità ad avere rapporti sessuali completi (patologie sessuologiche o problemi logistici)
  Sintomi algici anche gravi non saranno criteri di esclusione. Le pazienti con sintomi algici destinate alla FIVET saranno trattate per il dolore con terapia ormonale (progestinico, estroprogestinico o GnRH analogo) fino al tentativo. Queste terapie saranno sospese solo durante il periodo necessario allo svolgimento del ciclo FIVET. In caso di mancata risoluzione dei sintomi algici, la paziente verrà indirizzata a chirurgia ma verrà mantenuta nel braccio FIVET (analisi per “intention to treat”).

3.4. Criteri di inclusione dei controlli per analisi biologiche

- pazienti che hanno firmato il consenso informato
- indicazione alla FIVET
- assenza di endometriosi ovarica e peritoneale profonda.

3.5. Criteri di esclusione dei controlli per analisi biologiche

- Prelievo ovocitario per preservazione della fertilità

4. PROCEDURE RELATIVE ALLO STUDIO

4.1. Intervento
Alle pazienti eleggibili per lo studio verrà proposto di partecipare sottoponendo loro il consenso informato specifico. Accettando di partecipare, la paziente non potrà decidere autonomamente a quale delle due terapie sottoporsi (chirurgica o FIVET), per questo il medico dovrà spiegare alla paziente le due possibilità e discutere i rischi e i benefici di entrambe le procedure.
L’ecografia preliminare all’inclusione sarà eseguita esclusivamente da ecografisti esperti in forze nei centri partecipanti e sarà condotta secondo raccomandazioni internazionali (Guerriero et al., 2016).

Il trattamento chirurgico e i cicli FIVET saranno svolti secondo i protocolli clinici in vigore presso i vari Centri. La partecipazione allo studio non modificherà quanto fatto routinariamente.
Le pazienti sottoposte a chirurgia, successivamente all’intervento, riceveranno indicazioni per la ricerca di una gravidanza naturale da protrarsi fino a 12 mesi dal momento della randomizzazione, e saranno informate delle loro probabilità di gravidanza calcolate mediante il Endometriosis Fertility Index (EFI) (Adamson and Pasta, 2010), uno score validato appositamente per predire tale probabilità nelle pazienti endometriosiche Le donne non soddisfatte della prognosi prospettata potranno accedere prima del tempo alla FIVET.

Le pazienti che saranno incluse nel braccio FIVET saranno sottoposte a tre cicli completi di FIVET (ovvero tre prelievi ovocitari a prescindere dal numero di embryo transfer).
Alla visita –T1, si procederà a controllare i criteri di inclusione e lo studio e il suo razionale verrà spiegato alla paziente. Verrà consegnato e illustrato il consenso informato. Alle pazienti che si dimostreranno interessate verrà dato un appuntamento 1-2 settimane dopo (per consentirle di riflettere ed eventualmente chiedere ulteriori chiarimenti prima di iniziare) per la randomizzazione (T0). In tale occasione verranno compilati gli allegati A, B e C. Dopo la randomizzazione, le pazienti verranno messe in nota per l’intervento e/o la FIVET (classe B). Per le pazienti sottoposte all’intervento verrà compilato l’allegato E al controllo post-operatorio mentre per le donne nel braccio FIVET l’allegato F potrà essere compilato il giorno dell’embryo transfer (o del colloquio per mancato trasferimento nel caso non siano presenti embrioni vitali) e, nel caso di ottenimento della gravidanza, completato nelle visite successive. I controlli successivi saranno ogni 3 mesi (T1, T2 e T3) e consisteranno essenzialmente nell’accertarsi che il programma sia seguito nonché valutare la presenza di sintomatologia e l’eventuale insorgenza/evoluzione delle lesioni endometriosiche mediante ecografia TV. La visita finale (T4) sarà a 12 mesi dall’arruolamento. Nelle visite T1, T2, T3 e T4 dovranno essere compilati gli allegati B e C e, in caso di gravidanza, l’allegato . La pazienti che in quel momento saranno gravide verranno contattate 1-2 mesi dopo la data presunta del parto per accertarsi dell’esito della gravidanza e delle eventuali complicanze.

Una volta terminato il percorso (visita T4), le donne che non avranno ottenuto la gravidanza verranno consulentate riguardo l'opzione di cross-over, ovvero eseguire un intervento chirurgico per quelle assegnate alla FIVET e eseguire la FIVET per quelle programmate per l'intervento. Le pazienti che avranno intrapreso questo percorso verranno contattate telefonicamente ad un anno di distanza, (utilizzando risorse locali proprie non coperte dal finanziamento ministeriale), per un follow-up del trattamento, in particolar modo rispetto ad eventuali gravidanze insorte. Verrà infine effettuata un’ulteriore analisi di confronto su questi dati.

Le due strategie (chirurgia versus FIVET) differiscono radicalmente e, anche se le evidenze scientifiche non mostrano differenze di efficacia tra i trattamenti, le pazienti potrebbero essere riluttanti ad accettare l'allocazione casuale. Nel caso in cui questo causi difficoltà nel reclutamento e nella randomizzazione delle pazienti si valuterà se affiancare al presente studio un patient preference trial, richiedendone specifico parere al Comitato Etico.

Per l'obiettivo 3, il sangue periferico verrà raccolto da 50 donne del braccio FIVET e da un ugual numero di donne che si sottopongono a FIVET senza diagnosi di endometriosi abbinate in base a riserva ovarica (± 0.2 ng/ml in AMH), età (± 1 anno), dose iniziale di gonadotropine (stesso farmaco e identica dose) e periodo di studio. In questa parte di studio saranno coinvolte donne afferenti esclusivamente al Centro Coordinatore o all’Ospedale San Raffaele.

Analisi del liquido follicolare: i liquidi follicolari saranno raccolti durante il prelievo ovocitario senza che questo influenzi in alcun modo la metodica essendo essi un campione biologico “di scarto” che, dopo aver selezionato gli ovociti della donna, viene eliminato. Un campione di 1 ml di fluido follicolare privo di cellule dopo la centrifugazione verrà conservato a -80 ° C fino al dosaggio. La valutazione degli steroidi sarà effettuata mediante cromatografia e spettrometria di massa Sciex 5500 QTRAP (obiettivo 3b). L’analisi dei liquidi follicolari, raccolti in entrambi i Centri coinvolti in questa parte di studio, sarà eseguita sotto la responsabilità della dott.ssa Fustinoni presso la UOS Tossicologia Ambientale e Industriale del Centro Promotore

Analisi sui campioni plasmatici: per tutte le pazienti (endometriosiche e non) il prelievo ematico verrà effettuato in concomitanza dei prelievi routinariamente svolti durante le procedure per un volume di 10 ml. Le analisi dei campioni plasmatici raccolti in entrambi i Centri coinvolti in questa parte di studio saranno eseguite presso l’EPIGET Lab (Università degli Studi di Milano), sotto la responsabilità della Dott.ssa Bollati.

La frazione di plasma sarà utilizzata per purificare le vescicole extracellulari (EVs). Il plasma sarà centrifugato tre volte a 1000, 2000 e 3000 × g per 15 min a 4 ° C, e il pellet verrà scartato per rimuovere i detriti cellulari. Le vescicole verranno quindi concentrate mediante ultracentrifugazione a 110.000 × g per 120 min a 4 ° C. Per ottenere EVs intatte, i campioni non possono essere congelati e il plasma deve essere ulteriormente processato entro 3 giorni dal prelievo di sangue. L'analisi NTA verrà utilizzata per determinare i conteggi delle EV totali e le distribuzioni delle loro misure. Le EVs saranno caratterizzate dal punto di vista immunologico utilizzando la citofluorimetria a flusso MACSQuant Analyzer (Miltenyi Biotec, USA). Questa analisi delle EV è piuttosto impegnativa per le dimensioni limitate delle EVs. Per valutare l'integrità delle EVs, le aliquote dei campioni verranno colorate con carbossifluoresceina diacetato N-succinimidil estere (CFSE) a 37 ° C per 20 min al buio. CFSE è una molecola vitale non fluorescente che può entrare nelle EVs, dove le esterasi intracellulari rimuovono il gruppo acetato e convertono la molecola nella forma di estere fluorescente. Ogni aliquota di campione colorato con CFSE sarà quindi incubata con anticorpi specifici per indagare la possibile origine cellulare delle EVs: Ab-CD14 + (monociti / macrofagi), Ab-CD127 + / Ab-CD25 + (Tregs), Ab-CD4 + / Ab-CXCR3 + (Cellule Th1), Ab-CD4 + / Ab-CCR4 + (cellule Th2). L'analisi quantitativa dei dati di citofluorimetria a flusso sarà eseguita utilizzando il software FlowJo (Tree Star, Inc., Ashland, OR, USA) (obiettivo 3a2).
Dopo l'estrazione dei miRNA dalle EVs utilizzando i kit commerciali miRNeasy Mini e RNeasy MiniElute (Qiagen, Frederick, M , USA), l’miRNAome (754 miRNA) verrà analizzato con il sistema QuantStudio TM 12K Flex Real-Time PCR (Thermo Fisher) in un sottogruppo di campioni (i primi 25 casi e 25 controlli). Gli miRNA associati più strettamente all' infiammazione correlata all’endometriosi saranno ulteriormente confermati mediante real time PCR nel resto dei campioni (obiettivo 3a3).

Per il profilo proteomico, le EVs saranno processate presso il Functional Proteomic Lab di IFOM (servizio in service) in un sottogruppo di campioni (25 casi e 25 controlli). Le Evs saranno lisate, le proteine saranno quantificate e digerite. I campioni verranno caricati in duplicato su uno spettrometro di massa Orbitrap QExactive-HF quadrupolo nLC – ESI – MSMS (Thermo Fisher Scientific). I peptidi saranno separati su UHPLC Easy-nLC 1000 (Thermo Fisher Scientific) collegato a un emettitore di silice fusa di 25 cm con diametro interno di 75 μm (New Objective, Inc.), rivestito internamente con beads ReproSil-Pur C18-AQ 1.9 μm. I dati MS verranno acquisiti utilizzando un metodo top 15 in base ai dati per la frammentazione dell'HCD. Gli spettri MS a scansione completa (300–1650 Th) saranno acquisiti nell'Orbitrap con una risoluzione di 60 000, target AGC 3e6, IT 20 ms. Per gli spettri HCD, la risoluzione sarà impostata su 15 000 a m / z 200, target AGC 1e5, IT 80 ms; NCE 28% e larghezza di isolamento 1,2 m / z. Per la proteomica quantitativa, i file MS grezzi verranno elaborati con MaxQuant (versione 1.5.2.8) con database Uniprot_cp_human_setting. I peptidi e il FDR saranno impostati a 0,01; la lunghezza minima richiesta per un peptide sarà di sei aminoacidi e un minimo di due intensità di quantificazione (LFQ) sarà importato in Perseus (versione 1.5.0.31) applicando l'imputazione e la trasformazione del punteggio Z. L'analisi statistica sarà eseguita utilizzando il t-test, con correzione Benjamini – Hochberg, FDR 0,05 (obiettivo 3a4).

4.2. Randomizzazione
La randomizzazione verrà centralizzata in Policlinico: i pazienti arruolati saranno randomizzati utilizzando il modulo di randomizzazione REDCap. La randomizzazione avverrà in tre blocchi indipendenti corrispondenti ai tre Centri partecipanti.

5. ENDPOINT

5.1. Endpoint Primario
Probabilità di nati vivi da gravidanze iniziate entro 1 anno dal reclutamento confrontando la probabilità in donne randomizzate al braccio 1 (chirurgia) oppure al braccio 2 (FIVET).
5.2. Endpoint Secondari
Obiettivo 2) valutare se la procedura di FIVET sia più conveniente in termini di costo-efficacia rispetto alla chirurgia.
Obiettivo 3) comprendere se l'ambiente infiammatorio sistemico correlato all'endometriosi dimostrato dalla presenza di EVs circolanti caratterizzate da un profilo infiammatorio possa influenzare la qualità della follicologenesi e gli esiti della FIVET.
Obiettivo 4) probabilità di nati vivi per le pazienti che, non avendo ottenuto una gravidanza a seguito del trattamento, sceglieranno di eseguire il percorso di cross-over.

6. DURATA / TIMELINE DELLO STUDIO

Lo studio durerà tre anni a cui si aggiungerà un anno di follow up nel caso in cui le pazienti decidano di proseguire con il percorso di cross over. Il reclutamento terminerà entro un anno dall'inclusione della prima paziente. Lo studio inizierà una volta ottenuto parere favorevole dei comitati etici e la registrazione dello studio. Successivamente, i pazienti verranno seguiti fino alla fine del periodo di studio. Gli ultimi mesi saranno dedicati all'analisi e alla stesura dei papers.

Da 0 a 3 mesi: Comitato etico, identificazione del personale, attività di esercitazione
Da 4 a 15 mesi: reclutamento
Da 16 a 36 mesi: follow-up, raccolti dati, analisi biologiche, analisi dei dati e stesura articoli

scientifici sugli obiettivi da 1 a 3.
Da 16 a 48 mesi: follow up pazienti cross-over, raccolta e analisi dati e stesura articoli scientifici sull’obiettivo 4.
La pandemia di Covid-19 potrebbe rappresentare un ostacolo e causare un ritardo nell’inizio in quanto lo studio non potrà ovviamente iniziare fintantoché l’attività chirurgica ginecologica e di PMA dei Centri partecipanti non sia tornata alla normalità.

7. ANALISI STATISTICA

7.1. Dimensione del campione
Il calcolo della numerosità del campione si basa sui seguenti assunti: 1) percentuale attesa di gravidanza nel gruppo chirurgia pari a 30%, 2) errori tipo I e II di 0.05 e 0.20, rispettivamente 3) differenza in favore della FIVET che giustifichi i costi maggiori della procedura pari a 20% (i.e. percentuale di successo assoluto del 50%). Sulla base di queste premesse, il numero delle donne da randomizzare è 206 (103 per braccio). Considerando una percentuale di non-accettazione della randomizzazione intorno al 30%, il numero totale di donne da reclutare è 300. Il numero di pazienti per Centro dovrebbe essere simile (70-68-68) ma si adotterà una certa flessibilità (i centri che dovessero completare prima il reclutamento previsto localmente continueranno a reclutare fino al raggiungimento della numerosità totale prevista).
7.2. Analisi dei dati
I dati verranno analizzati utilizzando lo Statistical Package for Social Sciences (SPSS 26.0, IL, USA). Le analisi verranno eseguite con metodo Intention To Treat (ITT). Non è prevista un'analisi ad interim.
Verranno eseguite le seguenti analisi per i sottogruppi pre-specificati: centro di studio, stato di riserva ovarica (due gruppi in base ai livelli di mediana di AMH), età (< e > di 35 anni), analisi del seme (due gruppi basati sui livelli di mediana del numero totale di spermatozoi mobili), tipo di lesioni (endometriomi, lesioni peritoneali profonde o entrambi) e sintomi del dolore senza terapia medica (almeno un sintomo con scala di valutazione numerica > 5 vs nessuno). Le analisi si concentreranno esclusivamente sull'outcome primario (tasso di nati vivi).
I dati saranno presentati utilizzando il set di outcome principali recentemente suggerito per gli studi sull'infertilità (Duffy et al., 2019).

8. EVENTI AVVERSI

Il progetto non prevede somministrazione di farmaci o altre sostanze né pratiche cliniche al di fuori dello standard di cura solitamente proposto a queste pazienti. Gli eventi avversi che potranno verificarsi sono quindi quelli correlati all’effettuazione della terapia chirurgica o del ciclo FIVET e non si discosteranno da quanto atteso nella normale pratica clinica.

9. VALUTAZIONE RISCHIO / BENEFICIO

Lo studio non comporta rischi aggiuntivi per le pazienti trattandosi di pazienti che, a causa della presenza di endometriosi, sarebbero in ogni caso sottoposte ad uno dei due trattamenti.
Nello specifico i rischi connessi alla procedura FIVET possono essere visionati nel Consenso in uso presso UOSD PMA M.01.732.cons e quelli connessi al trattamento chirurgico nel Consenso in uso presso la UOC Ginecologia (M.18.719.CONS, M.16.719.CONS., M.29.719.CONS., M.29.719.CONS.all.01, M.30.719.CONS., M.30.719.CONS.All.1, M.21.719.CONS).

La paziente non avrà dalla partecipazione allo studio un effettivo beneficio considerando che i trattamenti utilizzati sono gli stessi che vengono proposti e discussi con le pazienti anche in assenza dello studio.
Lo studio avrà invece vantaggi immediati per il sistema sanitario nazionale (SSN). Considerando che l'obiettivo è identificare la migliore opzione per le donne infertili con endometriosi sia dal punto di vista clinico che economico, i risultati potrebbero essere utilizzati dal SSN per perfezionare le linee guida locali per la gestione dell'endometriosi e per arginare la grande eterogeneità di trattamenti oggi in atto sul territorio nazionale. Ancora più importante, sarà possibile consigliare le donne sulla base di dati robusti piuttosto che su considerazioni teoriche desunte da evidenze deboli (la mancanza di prove solide ha finora inevitabilmente esposto le pazienti a informazioni distorte da parte degli operatori sanitari). Inoltre, ci aspettiamo preziosi spunti dall'indagine sugli effetti dannosi mediati dall'infiammazione dell'endometriosi. In primo luogo, questa evidenza biologica potrebbe chiarire meglio i meccanismi di infertilità correlata all'endometriosi. In secondo luogo, potrebbe consentirci di identificare possibili bersagli per lo sviluppo di nuovi agenti terapeutici. Infine, potrebbero emergere nuovi biomarcatori di compromissione dell'infertilità, consentendo così di selezionare a priori donne che potrebbero essere trattate meglio con la chirurgia piuttosto che con la FIVET.

10. GESTIONE DELLO STUDIO

10.1. Raccolta e gestione dati
Ad ogni partecipante, al momento dell’arruolamento, verrà assegnato un codice univoco. Il file che associa il codice del partecipante con i relativi dati identificativi sarà conservato separatamente su un computer protetto da password. Il database dello studio sarà protetto da password e caricato su un computer anch’esso protetto da password ed accessibile al solo personale di studio designato dallo sperimentatore principale. La deidentificazione dei dati avverrà in maniera tale che le persone che accedono al database non potranno risalire in alcun modo all’identità dei soggetti. Solo gli sperimentatori locali potranno risalire all’identità dei soggetti arruolati.

La raccolta dati sarà centralizzata online mediante la CRF del Policlinico. I dati necessari per lo studio verranno registrati in una apposita eCRF in un Data Management System validato secondo la normativa nazionale, fornito dalla Direzione Scientifica della Fondazione. La piattaforma utilizzata sarà RedCap (Research Electronic Data Capture).

Il Consorzio REDCap è composto da >1000 partner istituzionali in tutto il mondo (enti di ricerca, università, ministeri etc). Il consorzio supporta un'applicazione web sicura (REDCap) progettato esclusivamente per supportare l'acquisizione di dati per studi di ricerca. L'applicazione REDCap consente agli utenti di creare e gestire banche dati on-line in modo rapido e sicuro, ed è attualmente in uso per più di 110.000 progetti con circa 150.000 utenti che coprono numerose aree di interesse di ricerca in tutto il consorzio.

Tramite REDCap, per questo studio verranno messi in atto: a) identificazione a livello di utente, con restrizioni specifiche in base al ruolo nello studio b) validazione e controllo dell’integrità dei dati in tempo reale c) de-identificazione dei pazienti prima dell’esportazione dei dati d) archiviazione centralizzata dei dati con backup giornaliero, un server sicuro all’interno della struttura informatica della Fondazione.

10.2 Aspetti regolatori e considerazioni etiche
10.2.1. Approvazione dell’Autorità Competente
In conformità con le normative vigenti, l’investigatore principale otterrà l'approvazione dall’appropriata Autorità Competente prima di iniziare lo studio clinico.
Questo studio sarà condotto in conformità con le regole dell'ICH / GCP (International Conference of Harmonization/Good Clinical Practice) e tutte le leggi applicabili, inclusa la Dichiarazione di Helsinki del giugno 1964, modificata dall'ultima World Medical Association General Assembly a Seoul, 2008.

10.2.2. Approvazione del Comitato Etico
Lo sperimentatore garantirà che il protocollo sia stato visto e approvato dal Comitato Etico indipendente locale (CE) prima di iniziare lo studio.
Il CE dovrà anche verificare e approvare il modulo di consenso informato (CI) e tutte le informazioni scritte ricevute dal paziente prima dell'arruolamento nello studio.
Qualora fosse necessario modificare il protocollo e / o il CI durante lo studio, lo sperimentatore sarà il garante e quindi la persona incaricata di garantire la revisione e l'approvazione di tale documento modificato secondo richiesta del CE. Il contenuto di tali modifiche sarà implementato solo dopo che il CE le avrà approvate. Fino a quel momento, sarà necessario fare riferimento alla versione precedente del documento già approvato.

10.2.3. Consenso informato (CI)

Lo sperimentatore o altro personale da lui incaricato ha il compito di informare le persone su tutti gli aspetti e le procedure dello studio.
Il processo per ottenere il consenso informato deve essere conforme alle procedure normative in vigore. L'investigatore (o un collaboratore designato) e il soggetto devono datare e firmare il modulo di consenso informato prima che il paziente avvii qualsiasi procedura relativa allo studio. Il soggetto riceverà una copia del CI datata e firmata da entrambe le parti; la copia originale sarà conservata negli archivi designati per lo studio. Né l'investigatore né il personale designato devono in alcun modo esercitare alcuna coercizione o influenza su un soggetto per indurlo a partecipare o continuare a partecipare allo studio. La decisione di un soggetto di partecipare allo studio deve essere completamente volontaria. L'investigatore e il personale designato devono sottolineare al soggetto che può revocare il proprio consenso in qualsiasi momento senza alcuna penalità o perdita di qualsiasi beneficio a cui possa avere diritto.

Le informazioni scritte o orali relative allo studio, compreso il modulo di consenso scritto, non contengono alcuna espressione linguistica che costringa il soggetto a rinunciare (anche solo apparentemente) ai suoi diritti legali, o che esoneri lo sperimentatore, l'ente o lo sponsor da responsabilità per negligenza.

10.3. Doveri dello sperimentatore
In conformità con le normative locali applicabili, lo sperimentatore invierà rapporti periodici riguardanti lo stato di avanzamento dello studio nel suo centro al CE e notificare allo stesso la chiusura dello studio. Le relazioni periodiche e la notifica di chiusura fanno parte delle responsabilità dello sperimentatore.

10.4. Monitoraggio dello studio
In conformità alle normative applicabili e la buona pratica clinica (GCP), il monitor visiterà o contatterà periodicamente il centro. La durata, la natura e la frequenza di tali visite / contatti dipendono dalla frequenza di reclutamento, dalla qualità dei documenti in possesso del centro e dalla loro adesione al protocollo. Verranno concertate con il monitor.

Attraverso tali contatti, il monitor deve:
• controllare e valutare l’avanzamento dello studio • esaminare i dati raccolti
• condurre la verifica del documento fonte
• identificare ogni problema e relative soluzioni

Gli scopi dell'attività di monitoraggio sono di verificare che:
• i diritti e il benessere del soggetto siano rispettati
• i dati dello studio siano accurati, completi e verificabili dai documenti originali
• lo studio sia condotto in conformità al protocollo ed eventuali emendamenti approvati, GCP e le normative applicabili

Lo sperimentatore deve:
• dare al monitor l'accesso diretto a tutta la documentazione pertinente
• dedicare parte del suo tempo e del suo staff al monitor per discutere i risultati del monitoraggio e ogni altro possibile aspetto.

Il monitor contatterà il centro prima dell'inizio dello studio per discutere il protocollo e le procedure di raccolta dei dati con lo staff.
In considerazione della lunghezza del follow-up (fino a due anni) e del periodo di reclutamento (12 mesi), non sono previste analisi ad interim (la quasi totalità dei pazienti reclutati sarà ancora in corso al momento della fine del reclutamento).

Eventuali decisioni sull’interruzione o modifiche del trial saranno prese di concerto tra il PI e tutti i partecipanti elencati a pagina 3-4. In caso di discordanza si procederà per votazione, in caso di parità prevarrà la decisione del PI.
Essendo lo studio finanziato dal Ministero della Salute (Grant di ricerca finalizzata), non sono previsti significativi conflitti di interesse. Il personale coinvolto nello studio ha però inevitabilmente personali conflitti di interesse che verranno raccolti e dichiarati al momento della redazione dello studio.

Il monitoraggio verrà dato in gestione a un monitor esterno indipendente. Le modalità verranno decise in occasione dell’incontro preliminare che avverrà prima del reclutamento del primo paziente.

10.5. Assicurazione della qualità dello studio
In qualità di promotore, Fondazione IRCCS Ca’ Granda, Ospedale Maggiore Policlinico può effettuare a propria discrezione un controllo sulla qualità dello studio. In questo caso, lo sperimentatore consentirà al monitor di accedere direttamente a tutta la documentazione pertinente e dedicare parte del suo tempo e del suo personale al revisore per discutere i risultati del monitoraggio ed eventuali altri aspetti dello studio.
Inoltre, le Autorità Regolatorie potranno eseguire ispezioni. In questo caso, lo sperimentatore autorizzerà all'ispettore l'accesso diretto a tutta la documentazione pertinente, e dedicherà parte del suo tempo e del suo personale all’ispettore stesso per discutere i risultati del monitoraggio ed eventuali altri aspetti dello studio.

10.6. Chiusura dello studio
Al momento della chiusura dello studio, il monitor e lo sperimentatore attiveranno una serie di procedure:

- rivedere tutta la documentazione dello studio
- riconciliare i dati dello studio
- conciliare tutte le relazioni di chiarimento.

10.7. Archiviazione di documenti
In conformità con le vigenti normative nazionali, lo sperimentatore conserverà una copia di tutta la documentazione in un luogo asciutto e sicuro dopo la chiusura dello studio (Per il Centro Coordinatore: Stanza 15, Centro PMA - Regina Elena).

10.8. Divulgazione di informazioni riguardanti la scoperta scientifica

10.8.1. Confidenzialità

Lo sperimentatore e altro personale coinvolto nello studio tratterà tutte le informazioni relative allo studio (compreso il protocollo, i dati ottenuti e tutta la documentazione prodotta nel corso dello studio) in modo confidenziale e non utilizzerà tali informazioni, dati o relazioni per scopi diversi da quelli descritti nel protocollo. Queste restrizioni non si applicano a:
1) informazioni che diventano pubblicamente disponibili, non a causa di negligenza da parte dello sperimentatore o del suo personale;
2) informazioni che richiedono la divulgazione riservata a CE al solo scopo di valutare lo studio;
3) informazioni che devono essere divulgate al fine di ottenere cure mediche adeguate per un soggetto di studio.

10.8.2. Pubblicazioni

I responsabili scientifici dello studio si impegneranno nella stesura di un rapporto finale e a rendere pubblici i risultati al termine dello studio. I dati saranno resi pubblici in modo anonimo e presentati per quanto richiesto in modalità aggregata (i dati potranno però essere inviati alla rivista scientifica in forma non-aggregata e in modo deidentificato, se specificatamente richiesto). I risultati saranno disseminati tramite comunicazioni a congressi nazionali ed internazionali e pubblicazioni su riviste scientifiche seguendo le linee guida internazionali di definizione dell’authorship. In caso di pubblicazioni disgiunte, ciascuna pubblicazione dovrà menzionare che tali risultati sono stati raggiunti nell’ambito del presente Progetto.

10.9. Diritti di proprietà intellettuale sui risultati dello studio
Il Promotore dello Studio e i Centri Partecipanti si danno reciprocamente atto sin da ora che nel corso dell’attuazione del Progetto, nei limiti di quanto strettamente necessario allo svolgimento di questo, potrebbero essere utilizzati in varia misura know-how, materiale tecnico e/o beni protetti da diritti di proprietà industriale e/o intellettuale o suscettibili di protezione, sviluppati prima dell’avvio del Progetto dal Promotore e dai Centri Partecipanti che restano nella titolarità di ciascuna di esse e su cui non si acquisirà alcun diritto di qualsivoglia tipo.
I risultati delle attività di ricerca resteranno di proprietà del Promotore e dei Centri Partecipanti proporzionalmente al rispettivo contributo inventivo e la loro utilizzazione, restando inteso che la messa a disposizione di campioni biologici alla base del progetto di ricerca, delle informazioni cliniche correlate e del relativo know-how medicale da parte del Promotore dello studio verranno considerate quale un contributo essenziale. In caso di risultati innovativi, suscettibili di protezione brevettuale (o simile titolo di privativa) e/o sfruttamento economico, la parti contitolari si impegnano a regolare in uno specifico accordo le modalità di tutela e valorizzazione di detti risultati. In questo caso, le eventuali pubblicazioni saranno subordinate all’espletamento di tutte le procedure atte alla protezione brevettuale dei risultati.

11. INDENNITÀ E RISARCIMENTI IN CASO DI DANNI

In caso di eventi indesiderati o eventuali danni che dovessero derivare dalla partecipazione alla ricerca, la Polizza Assicurativa del nostro Istituto è estesa anche alla copertura dei soggetti partecipanti ai progetti di ricerca.

12. EMENDAMENTI AL PROTOCOLLO

Eventuali modifiche al protocollo verranno tempestivamente richieste al Comitato Etico competente.

13. ACCORDI FINANZIARI

Il presente progetto è stato finanziato dal Ministero della Salute – Bando Ricerca Finalizzata 2019.

14. DISCLOSURE SUI CONFLITTI DI INTERESSE

Vedi modulo allegato.

15. REFERENZE

Adamson GD, Pasta DJ. Endometriosis fertility index: the new, validated endometriosis staging system. Fertil Steril. 2010 Oct;94(5):1609-15.

Bafort C, Beebeejaun Y, Tomassetti C, Bosteels J, Duffy JM. Laparoscopic surgery for endometriosis. Cochrane Database Syst Rev. 2020 Oct 23;10:CD011031.

Duffy JMN. COMMIT: Developing a core outcome set for infertility research. Hum Reprod. Abstract book 2019; 34 Suppl. 1, pp 171, O-218.
Accessible at: https://cm.eshre.eu/presentations/ESHRE2019/O-219/default.aspx

ETIC Endometriosis Treatment Italian Club. When more is not better: 10 'don'ts' in endometriosis management. An ETIC position statement. Hum Reprod Open. 2019 Jun 12;2019(3):hoz009.

Guerriero S, Condous G, van den Bosch T, Valentin L, Leone FP, Van Schoubroeck D, Exacoustos C, Installé AJ, Martins WP, Abrao MS, Hudelist G, Bazot M, Alcazar JL, Gonçalves MO, Pascual MA, Ajossa S, Savelli L, Dunham R, Reid S, Menakaya U, Bourne T, Ferrero S, Leon M, Bignardi T, Holland T, Jurkovic D, Benacerraf B, Osuga Y, Somigliana E, Timmerman D. Systematic approach to sonographic evaluation of the pelvis in women with suspected endometriosis, including terms, definitions and measurements: a consensus opinion from the International Deep Endometriosis Analysis (IDEA) group. Ultrasound Obstet Gynecol. 2016 Sep;48(3):318-32.

Harb HM, Gallos ID, Chu J, Harb M, Coomarasamy A. The effect of endometriosis on in vitro fertilisation outcome: a systematic review and meta-analysis. BJOG. 2013 Oct;120(11):1308-20.

Hodgson RM, Lee HL, Wang R, Mol BW, Johnson N. Interventions for endometriosis-related infertility: a systematic review and network meta-analysis. Fertil Steril. 2020 Feb;113(2):374- 382.e2.

Nisenblat V, Bossuyt PM, Farquhar C, Johnson N, Hull ML. Imaging modalities for the non- invasive diagnosis of endometriosis. Cochrane Database Syst Rev. 2016 Feb 26;2(2):CD009591.

Somigliana E, Benaglia L, Paffoni A, Busnelli A, Vigano P, Vercellini P. Risks of conservative management in women with ovarian endometriomas undergoing IVF. Hum Reprod Update. 2015 Jul-Aug;21(4):486-99

Somigliana E, Vigano P, Benaglia L, Busnelli A, Berlanda N, Vercellini P. Management of Endometriosis in the Infertile Patient. Semin Reprod Med. 2017 Jan;35(1):31-37.

Somigliana E, Viganò P, Benaglia L, Busnelli A, Paffoni A, Vercellini P. Ovarian stimulation and endometriosis progression or recurrence: a systematic review. Reprod Biomed Online. 2019 Feb;38(2):185-194.

Vercellini P, Somigliana E, Viganò P, Abbiati A, Barbara G, Crosignani PG. Surgery for endometriosis-associated infertility: a pragmatic approach. Hum Reprod. 2009 Feb;24(2):254-69.

Vercellini P, Barbara G, Buggio L, Frattaruolo MP, Somigliana E, Fedele L. Effect of patient selection on estimate of reproductive success after surgery for rectovaginal endometriosis: literature review. Reprod Biomed Online. 2012 Apr;24(4):389-95.

Vercellini P, Viganò P, Somigliana E, Fedele L. Endometriosis: pathogenesis and treatment. Nat Rev Endocrinol. 2014 May;10(5):261-75.

Vercellini P, Viganò P, Frattaruolo MP, Borghi A, Somigliana E. Bowel surgery as a fertility- enhancing procedure in patients with colorectal endometriosis: methodological, pathogenic and ethical issues. Hum Reprod. 2018 Jul 1;33(7):1205-1211.

WHO laboratory manual for the examination and processing of human semen Fifth edition, 2010 Available at: https://www.who.int/reproductivehealth/publications/infertility/9789241547789/en/
